# Unraveling essential cellulosomal components of the (*Pseudo*)*Bacteroides cellulosolvens* reveals an extensive reservoir of novel catalytic enzymes

**DOI:** 10.1186/s13068-019-1447-2

**Published:** 2019-05-09

**Authors:** Olga Zhivin-Nissan, Bareket Dassa, Ely Morag, Meital Kupervaser, Yishai Levin, Edward A. Bayer

**Affiliations:** 1 Department of Biomolecular Sciences, The Weizmann Institute of Science, Rehovot, Israel; 2 Bioinformatics Unit, Life Sciences Core Facilities, Weizmann Institute of Science, Rehovot, Israel; 3 Proteomics Unit, Nancy and Stephen Grand Israel National Center for Personalized Medicine, Weizmann Institute of Science, Rehovot, Israel

**Keywords:** Biotechnology, Cohesin, Dockerin, Cellulolytic bacteria, CBM, Cellulases, Enzymatic hydrolysis, Glycoside hydrolases, Scaffoldin

## Abstract

**Background:**

(*Pseudo*)*Bacteroides cellulosolvens* is a cellulolytic bacterium that produces the most extensive and intricate cellulosomal system known in nature. Recently, the elaborate architecture of the *B. cellulosolvens* cellulosomal system was revealed from analysis of its genome sequence, and the first evidence regarding the interactions between its structural and enzymatic components were detected in vitro. Yet, the understanding of the cellulolytic potential of the bacterium in carbohydrate deconstruction is inextricably linked to its high-molecular-weight protein complexes, which are secreted from the bacterium.

**Results:**

The current proteome-wide work reveals patterns of protein expression of the various cellulosomal components, and explores the signature of differential expression upon growth of the bacterium on two major carbon sources—cellobiose and microcrystalline cellulose. Mass spectrometry analysis of the bacterial secretome revealed the expression of 24 scaffoldin structural units and 166 dockerin-bearing components (mainly enzymes), in addition to free enzymatic subunits. The dockerin-bearing components comprise cell-free and cell-bound cellulosomes for more efficient carbohydrate degradation. Various glycoside hydrolase (GH) family members were represented among 102 carbohydrate-degrading enzymes, including the omnipresent, most abundant GH48 exoglucanase. Specific cellulosomal components were found in different molecular-weight fractions associated with cell growth on different carbon sources. Overall, microcrystalline cellulose-derived cellulosomes showed markedly higher expression levels of the structural and enzymatic components, and exhibited the highest degradation activity on five different cellulosic and/or hemicellulosic carbohydrates. The cellulosomal activity of *B. cellulosolvens* showed high degradation rates that are very promising in biotechnological terms and were compatible with the activity levels exhibited by *Clostridium thermocellum* purified cellulosomes.

**Conclusions:**

The current research demonstrates the involvement of key cellulosomal factors that participate in the mechanism of carbohydrate degradation by *B. cellulosolvens.* The powerful ability of the bacterium to exhibit different degradation strategies on various carbon sources was revealed. The novel reservoir of cellulolytic components of the cellulosomal degradation machineries may serve as a pool for designing new cellulolytic cocktails for biotechnological purposes.

**Electronic supplementary material:**

The online version of this article (10.1186/s13068-019-1447-2) contains supplementary material, which is available to authorized users.

## Background

The rising demand for renewable alternative fuels derives from our rapidly growing, global population and concerns about climate change and environmental pollution [1]. Biofuels are produced from biological materials, mainly renewable plant biomass [2]. Biofuels are a current practical solution to the global energy problem and are a promising strategy for future decarbonization. Biomass encompasses material that originates from woody, herbaceous and aquatic plants [3]. Massive amounts of cellulose are also accessible in form of industrial and municipal wastes, which aggravate pollution problems and thus increase our interest to convert cellulosic biomass to bioethanol.

Cellulose is the most abundant renewable organic compound on earth [4]. Aside from being the primary building material for plants, cellulose has many other uses. Cellulose is a highly polymerised homopolysaccharide. One of the most efficient ways of cellulose degradation was “invented” by cellulolytic microbes, and some anaerobic bacteria secrete a multiprotein cellulosomal complex capable of deconstruction of cellulose and associated plant wall polysaccharides [5, 6]. Cellulosomal enzymes, capable of synergistic action and physical proximity to the insoluble substrate, are organized into large complexes via structural scaffoldin subunits [7]. The scaffoldins possess one or more cohesin modules, which interact with dockerin-bearing enzymatic or scaffoldin subunits to form one of the strongest protein–protein interactions found in nature [8, 9]. Following the original discovery in *Clostridium thermocellum* [5], cellulosomal systems have been found in other bacteria. Currently there are 19 known species of cellulosome-producing bacteria (both mesophilic and thermophilic) [6]. Some of these bacteria, e.g., *Clostridium cellulolyticum*, *Clostridium josui*, and *C. papyrosolvens*, produce simple cellulosomal systems with a single major scaffoldin that bears only type I cohesins for integration of the dockerin-containing enzymes. Others, e.g., *C. thermocellum*, *C. clariflavum*, and *Acetivibrio cellulolyticus*, produce complex cellulosomal systems, in which primary scaffoldins bear type I cohesins whereas a second class of scaffoldin contains type II cohesins that anchor the cellulosome to the bacterial cell surface [6].

Carbohydrate composition and structure differ among different types of plant cell wall species. Lignocellulosic biomass usually undergoes a pre-treatment step(s) in order to facilitate the degradation process and modify biomass composition [10, 11]. Consequently, different compositions of enzymatic complexes should be used for the type (content) of biomass to be degraded. In order to create strategies for efficient biomass conversion and design ways for effective enzymatic degradation, we need to understand the metabolic potential of the different bacterial species. Proteomics could therefore provide insights into the selection of specific enzymes for degradation of defined carbohydrates [12–14]. It was shown previously that *C. thermocellum* can adjust cellulosome content in order to fulfill its growth requirements [15]. In this context, the bacterium senses the biomass composition in the medium and controls the composition of cellulosomal components to suit the requirements for degradation of the specific carbohydrates [16–21]. Proteomic studies are important thus enabling us to understand the role of the biomass in cellulosomal regulation and allowing us to elucidate the key enzymes participating in efficient degradation [12, 15, 22].

The current research concentrates on proteomic examination of (*Pseudo*)*Bacteroides cellulosolvens*—a mesophilic, anaerobic, cellulosome-producing bacterium capable of growing on cellobiose and cellulose as sole carbon sources. The bacterium was originally classified as *B*. *cellulosolvens* [23] but later found to be phylogenetically related to the clostridial assemblage [24] and more recently reclassified as *Pseudobacteroides cellulosolvens* [25]. For the purposes of the present work, we will continue to refer to the original name. In our previous research on the cellulosome system of this bacterium [26], we performed a complete bioinformatic analysis of the bacterial genome and revealed a remarkable number of cellulosomal elements, including 32 scaffoldins with 79 cohesins and 212 dockerin-bearing ORFs. The cellulosomal arrangement in this bacterium is distinct in comparison to other cellulosomal systems. The types of the cohesins are reversed in all *B. cellulosolvens* scaffoldins, namely the primary scaffoldins that incorporate enzymes bear type II cohesins whereas the type I cohesins are positioned on the anchoring scaffoldins. This is opposed to all previously described complex cellulosomal systems, notably that of *C. thermocellum*, where the primary scaffoldins possess type I cohesins and the anchoring scaffoldins contain type II cohesins.

Here, we present a first proteome-wide study of *B. cellulosolvens*, which unravels the diverse architecture and complexity of its cellulolytic enzymatic pool. We investigated the extracellular proteome of *B. cellulosolvens,* grown on two different cellulosic substrates: the soluble disaccharide cellobiose and the insoluble polymeric microcrystalline cellulose (Avicel). Comparison of the extracellular proteomic profile between the substrates assisted our comprehension of the significance and cellulolytic potential of *B. cellulosolvens,* in carbohydrate deconstruction towards cellulosic ethanol production.

## Methods

### Bacterial strains

*Bacteroides cellulosolvens* DSM 2933 and *C. thermocellum* DSM 1313 were purchased from the Leibniz Institute DSMZ (German Collection of Microorganisms and Cell Cultures, Braunschweig, Germany).

### Anaerobic fermentation

*Bacteroides cellulosolvens* was grown on 315 medium (DSMZ) containing (per liter distilled water): 0.68 g NH_4_Cl, 0.30 g K_2_HPO_4_, 0.18 g KH_2_PO_4_, 0.15 g (NH_4_)_2_SO_4_, 0.12 g MgSO_4_ × 7H_2_O, 0.06 g CaCl_2_ × 2H_2_O, 0.02 g FeSO_4_ × 7H_2_O, 10 ml trace element solution (see below), 10 ml BME vitamin solution (Sigma), 5 g cellobiose or 5 g cellulose, 1 mg resazurin, 2 g NaHCO_3_, 0.25 g cysteine-HCl × H_2_O, and 0.25 g Na_2_S × 9H_2_O. Trace element solution (per liter distilled water): 1.5 g nitrilotriacetic acid, 3 g MgSO_4_ × 7H_2_O, 0.5 g MnSO_4_ × H_2_O, 1 g NaCl, 0.1 g FeSO_4_ × 7H_2_O, 0.18 g CoSO_4_ × 7H_2_O, 0.1 g CaCl_2_ × 2H_2_O, 0.18 g ZnSO_4_ × 7H_2_O, 0.01 g CuSO_4_ × 5H_2_O, 0.02 g KAl(SO_4_)_2_ × 12H_2_O, 0.01 g H_3_BO_3_, 0.01 g Na_2_MoO_4_ × 2H_2_O, 0.025 g NiCl_2_ × 6H_2_O, 0.3 mg Na_2_SeO_3_ × 5H_2_O. The bacterium was grown at 35 °C, pH 7.2. Cellobiose (MP Biomedicals, Illkirch, France, 5 g/l) or microcrystalline cellulose (Avicel; Sigma-Aldrich, 5 g/liter) served as a carbon source during fermentation in 5 l glass fermentors. Growth on each of the two carbon sources was performed in three biological repeats. The bacterial cells were harvested at the stationary phase, the supernatant was filtered through sterile plastic filters (Thermo, Fisher Scientific, Waltham, MA, USA) and concentrated 100-fold, using a peristaltic pump (MasterFlex l/S pump system, Easy-Load II pump head [Cole-Parmer, Vernon Hills, IL]) with a 300-kDa-cutoff Pellicon 2 membrane (Millipore, Darmstadt, Germany).

During fermentation, bacterial growth was examined by measuring protein content, OD_600_ (in soluble cellobiose-grown cultures), NaOH consumption in order to stabilize the pH level, and CMCase (carboxymethyl cellulose, CMC; VWR International Ltd., Poole, England) activity to gauge the presence of catalytic enzymes. CMCase activity tests of the supernatant fluids were measured by the dinitrosalicylic acid (DNS) assay and [27] were carried out to estimate the level of the secreted cellulolytic enzymes and growth phases. Indeed, the activity reached its highest point at the stationary phase of growth, at which the fermentors were harvested. Cellobiose-grown cultures reached stationary phase after 40–48 h, while cellulose-grown cells reached the same state after 60 h. The general protein amount also increased over time (total protein concentration measurements were taken with bicinchoninic acid assay [28] [BCA protein kit, Thermo Scientific, Rockford, USA]). According to the increasing activity results, we assume that the total protein concentration increased, due to elevated secretion of cellulosomal proteins as previously reported [29].

### Isolation of high-molecular-weight complexes

Prior to the isolation step, CMCase activity of concentrated fractions was measured by the dinitrosalicylic acid (DNS) assay [27], in order to ensure the presence of cellulolytic complexes. High-molecular-weight complexes were isolated by gel filtration chromatography using a preparative chromatography system for laboratory-scale protein purification (Äkta start; GE Healthcare, Uppsala, Sweden). The samples were loaded onto a Superose 6 Increase gel filtration column (GE Healthcare) with Tris-buffered saline as the running buffer (TBS; 137 mM NaCl, 2.7 mM KCl, 25 mM Tris–HCl [pH 7.4]). Two major peaks were obtained during the gel filtration process. Examination of the peaks revealed two different populations of high-molecular-weight protein complexes that were active on CMC. Fractions within each peak were pooled together and concentrated with a Vivaspin concentrator (20 ml tubes with 50-kDa-cutoff membrane; Sartorius Stedim Biotech GmbH, Göttingen, Germany). Protein concentrations were measured by the bicinchoninic acid (BCA) assay.

### *C. thermocellum* cellulosome purification

*Clostridium thermocellum* cellulosomes were grown on microcrystalline cellulose and prepared according to Yoav et al. [30].

### β-Glucosidase expression and purification

A pET28a cassette, containing the His-tagged wild-type (WT) bglC gene from the *Thermobifida fusca* genome was obtained from Dr. David B. Wilson [31]. The plasmid was transformed into *Escherichia coli* BL21, and the cells were grown in 1 l of Luria–Bertani broth (LB), containing 50 µg/ml kanamycin, for 2 h at 37 °C to an A_600_ of ~ 0.8. Isopropyl-1-thio-β-d-galactoside (IPTG; 0.2 mM) (Fermentas UAB, Vilnius, Lithuania) was added to induce protein expression. Cells were incubated for an additional 18 h at 16 °C. Cells were harvested (4000*g*, 15 min) in Sorval RC6 Plus centrifuge (Thermo) and sonicated, then centrifuged (20,000*g*, 30 min). The protein was purified on nickel–nitrilotriacetic acid (Ni–NTA) beads in a batch purification system as described previously [32]. Protein concentration was determined by absorbance at 280 nm and evaluated based on the extinction coefficient, calculated using the Expasy ProtParam tool (http://web.expasy.org/protparam/). The protein was stored in 50% (vol/vol) glycerol at − 20 °C.

### Sample preparation for mass spectrometry analysis

Bacterial growth media was concentrated on a 3 kDa MwCO filter (Merck, Darmstadt, Germany), the buffer exchanged to 8 M urea (Sigma-Aldrich, U5128) in 0.1 M Tris–HCl, pH 7.9, and the protein concentration measured. Protein samples (50 μg) were first reduced by incubation with dithiothreitol (5 mM; Sigma-Aldrich) for 1 h at room temperature, and alkylated with 10 mM iodoacetamide (Sigma-Aldrich) in the dark for 45 min. The sample was diluted to 2 M urea with 50 mM ammonium bicarbonate. Proteins were then subjected to digestion with trypsin (Promega; Madison, WI) overnight at 37 °C (50:1 protein amount: trypsin), followed by a second trypsin digestion for 4 h. The digestions were stopped by the addition of trifluoroacetic acid (1%). Following digestion, peptides were desalted on solid-phase extraction columns (Oasis HLB, Waters, Milford, MA, USA) and stored in − 80 °C until further analysis.

### Liquid chromatography

ULC/MS grade solvents were used for all chromatographic steps. Each sample was loaded using split-less nano-Ultra Performance Liquid Chromatography (10 kpsi nanoAcquity; Waters, Milford, MA). The mobile phase was: A: H_2_O + 0.1% formic acid and B: acetonitrile + 0.1% formic acid. Desalting of the samples was performed online using a reversed-phase C18 trapping column (180 μm internal diameter, 20 mm length, 5 μm particle size; waters). The peptides were then separated using a T3 HSS nano-column (75 μm internal diameter, 250 mm length, 1.8 μm particle size; waters) at 0.35 μl/min. Peptides were eluted from the column into the mass spectrometer using the following gradient: 4% to 20% B in 155 min, 20% to 90% B in 5 min, maintained at 90% B for 5 min and then back to initial conditions.

### Mass spectrometry

The nanoUPLC was coupled online through a nanoESI emitter (10 μm tip; New Objective; Woburn, MA) to a quadrupole orbitrap mass spectrometer (Q Exactive HF, Thermo Scientific) using a FlexIon nanospray apparatus (Proxeon).

Data were acquired in DDA mode, using a Top20 method. MS1 resolution was set to 120,000 (at 400 *m*/*z*), and maximum injection time was set to 20 ms. MS2 resolution was set to 60,000 and maximum injection time of 60 ms.

### Data processing and bioinformatic analysis

Raw data were processed using MaxQuant v1.6.0.16. MS/MS spectra were searched using MaxQuant’s built-in search engine, Andromeda. Data were searched against the *Pseudobacteroides cellulosolvens* ATCC 35603 DSM 2993 sequences in UniprotKB (Additional file 1: Table S1). Fixed modification was set to carbamidomethylation of cysteines, and variable modifications were set to oxidation of methionines and deamidation of glutamine and asparagine. Protein identifications were filtered, such that the global false discovery rate was maximum of 1%. Comparative analysis of LFQ intensities was done in Perseus (v1.6.0.7) to determine fold changes and *p*-values, adjusted with multiple-comparison correction. Proteins resulting in the MaxQuant file of tryptic digestion were filtered to remove reverse sequences and known mass spectrometry contaminants. Protein annotation was based on the CAZy database (http://www.cazy.org/) and a previous publication of the *P. cellulosolvens* genome [26, 33]. Unsupervised hierarchical clustering was done using the Euclidian method with average linkage. The resulting heatmaps and PCA projection [34] were generated using the Partek Genomics Suite software, version 7.0. The mass spectrometry proteomics data have been deposited to the ProteomeXchange Consortium via the PRIDE (http://www.ebi.ac.uk/pride) partner repository with the dataset identifier PXD012663.

### Activity assays

Activity assays were performed in a total volume of 500 µl, containing 50 mM acetate buffer (pH 6), 12 mM CaCl_2_, 2 mM EDTA, and 50 µg of each cellulosome complex. The activity of *B. cellulosolvens* high-molecular-weight complexes was tested on five cellulosic substrates: Avicel (microcrystalline cellulose, 7.5 mg/ml, 24 h at 40 °C); Xylan (1% of beechwood xylan [Sigma-Aldrich, Rehovot, Israel], 1 h at 40 °C); carboxymethyl cellulose (CMC, 1%, for 1 h at 40 °C); phosphoric acid-swollen cellulose (PASC, was assayed at a final concentration of 5.6 mg/ml, 3 h at 40 °C); wheat straw (5 mg/ml alkaline-pretreated, 24 h at 40 °C). Preparations of PASC and wheat straw are detailed below. All degradation assays included *C. thermocellum* cellulosome, used as a positive control, which was incubated at 60 °C at similar time intervals. *T. fusca* β-glucosidase (BglC) was added at concentration of 15 µg/ml. All experiments were performed in duplicates three times in 2-ml tubes. Tubes were incubated with shaking. The reaction was terminated by flash-cooling the tubes on ice followed by centrifugation (22,000*g*, 5 min). Samples (100 µl) were transferred into 150 µl dinitrosalicylic acid (DNS) solution. The tubes were boiled for 10 min at 100 °C, and absorbance was measured at 540 nm in 96-well plates in a plate reader. The enzymatic activity was evaluated by calculating the concentration (millimolar) of released reducing sugars according to a glucose standard curve for determining the amount of reducing sugars.

### Wheat straw preparation

Hatched wheat straw (0.2–0.8 mm), purchased from Valagro (Poitiers, France), was washed as described earlier [35, 36] and treated for 1 h with 12% sodium hypochlorite at room temperature [36]. The goal of this treatment was to decrease the lignin concentration while keeping the cellulose and hemicellulose concentrations stable. Following pre-treatment, the wheat straw was washed in distilled water until no sodium hypochlorite residues were detected (according to the pH measurements) and vacuum filtered on a 2.7-μm glass filter. The concentration of the residual material was estimated by dry weight.

### PASC preparation

Avicel (12 g) was stirred in 0.5 l double-distilled water (DDW) until a homogeneous suspension was obtained. Concentrated phosphoric acid (600 ml) was then added, and the suspension was incubated for 2 h with stirring in a hood at room temperature, followed by addition of 3 l DDW, centrifugation at 15,000*g* for 35 min. The precipitate was then resuspended in DDW, and brought to pH 7 by titration with NaOH.

## Results

### Purification and fractionation of secreted cellulosome complexes

**Fig. 1:**
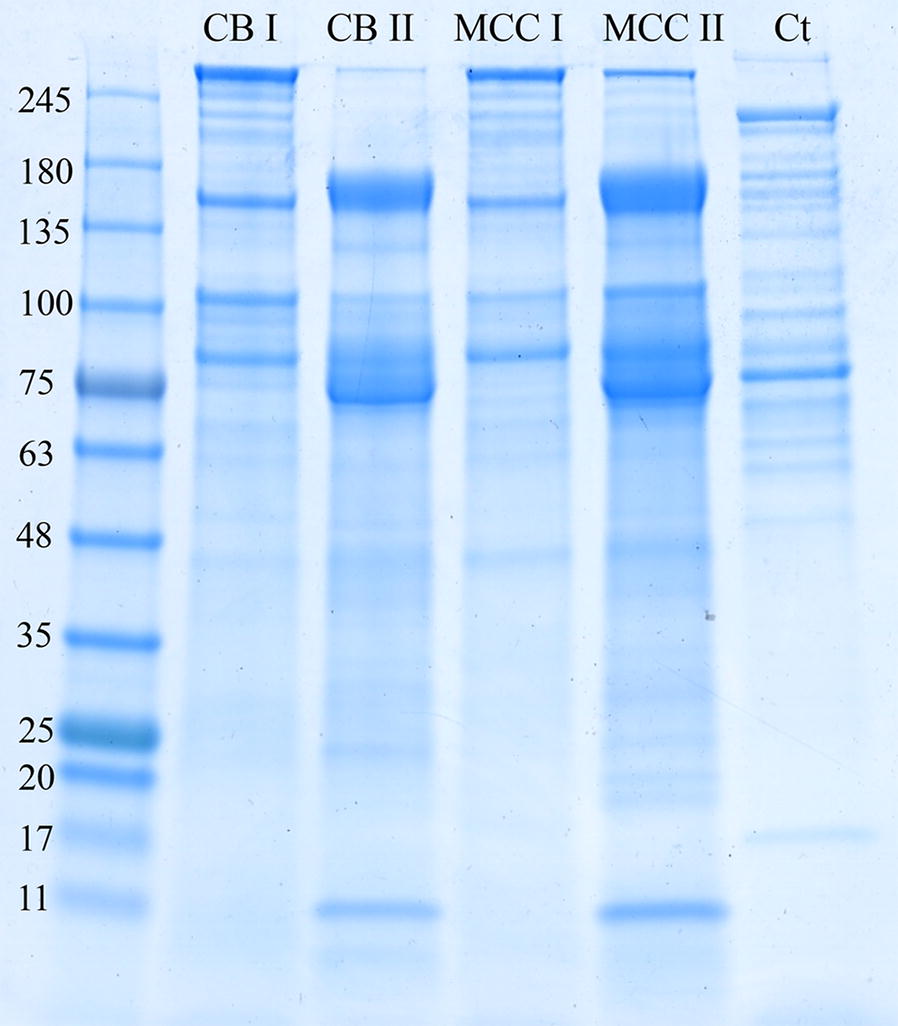
SDS-PAGE analysis of the high-molecular-weight cellulosomal fractions. *B. cellulosolvens* cellulosomal fractions, derived from cellobiose- and microcrystalline cellulose-grown cells, were separated by gel filtration (see Additional file 2: Figure S1). Each spent-cell medium (concentrated supernatant fluids) is represented by two peaks—I and II. The cellulosomes (20 µg) were subjected to 4-to-15% gradient SDS-PAGE. CB: cellobiose; MCC: microcrystalline cellulose; Ct: purified *C. thermocellum* cellulosome

In order to evaluate the proteomic composition of *B. cellulosolvens* cellulosomes, we purified the extracellular medium of *B. cellulosolvens* cells, after anaerobic growth of the bacterium on two types of carbon source: cellobiose (CB) and microcrystalline cellulose (MCC). After harvesting the cultures at the highest level of catalytic activity (stationary phase), supernatant fluids were collected and concentrated (300-kDa-cutoff), in order to separate high-molecular-weight protein complexes. The extracellular protein content within the concentrated fractions was further separated by gel filtration. Two major high-molecular-weight peaks were observed in each carbon source (Fractions I and II, Additional file 2: Figure S1). The collected fractions of each peak were separated by SDS-PAGE, and the protein population of each peak was assessed (Fig. 1). The fractions within the peaks were pooled according to similarity of their protein profiles and the presence of CMCase activity. The first eluted peak represented higher molecular-weight protein complexes (Fraction I), and the second peak represented lower-molecular-weight protein complexes or free proteins (Fraction II). SDS-PAGE examination of isolated fractions generated a similar profile of cellulosomal components between high-molecular-weight fractions of cellobiose (CB_I) and microcrystalline cellulose (MCC_I) as well as between the lower-molecular-weight fractions for both substrates (CB_II and MCC_II, respectively). Comparison of the cellulosome profiles of *B. cellulosolvens* and *C. thermocellum* revealed different patterns of protein content, indicating significant differences in the population of enzymes and structural proteins of the two species.

### Distinctive proteomic profiles of high-molecular-weight cellulosomal fractions

Proteins in the two molecular-weight fractions (Fractions I and II), resulting from growth of *B. cellulosolvens* cells on the two different carbon sources, were subjected to mass spectrometry (MS) analysis. This resulted in 1510 proteins, of which the similarity and variation between the protein samples were examined further by analyzing their intensities.

**Fig. 2:**
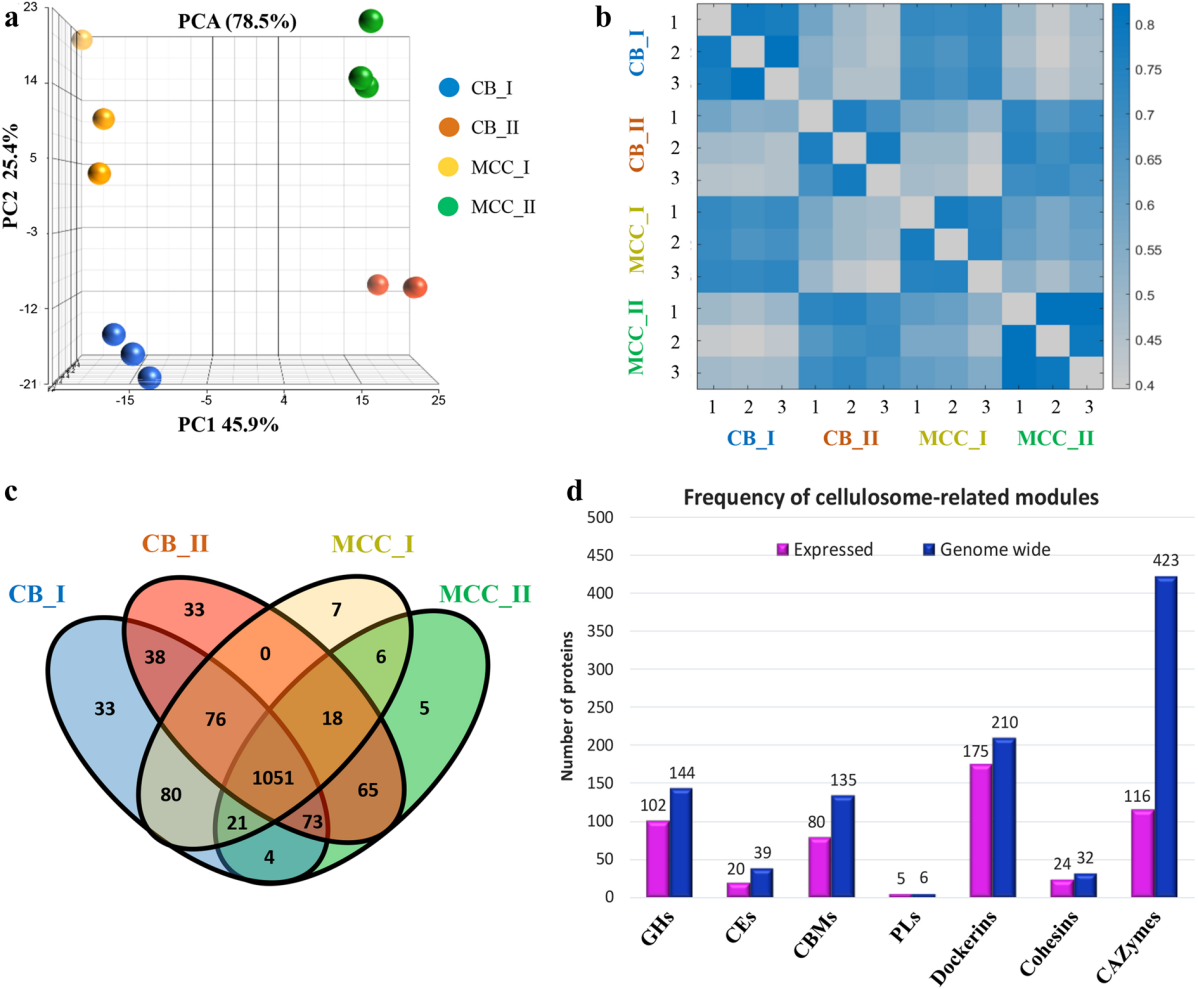
Proteomic profiling of the cellulosomal fractions. **a** Principal component analysis (PCA), for estimating the variance between all samples, showed a separation between the protein expression profiles of the two peak fractions (I or II), and also between proteins originating from cells grown on either MCC (microcrystalline cellulose) or CB (cellobiose). The PC1 axis is the first principal direction, along which the samples show the largest variation, and the PC2 axis is the second principle component. Percentage of the variance contributed by each principal component is indicated in the axis. **b** Pearson correlation coefficients for each pairwise combinations of samples (calculated from log2 LFQ values). High correlation was detected within the replicates (1–3) and also within replicates of the same peak (I or II). **c** Venn diagram depicting the overlap in the number of proteins, which were detected in replicates of the samples and/or between the different carbon sources. **d** Number of proteins containing CAZy- and cellulosome-related modules, which were detected among the 1510 proteins identified in this study (detectable in the secretome, not necessarily differentially expressed above a certain threshold). Magenta—proteins detected in this study, blue—proteins coded in the genome. Most of the cellulosomal modules are expressed. Full list of protein names and intensities is given in Additional file 1: Table S1

Principle component analysis (PCA) [34] was applied to identify variations between the samples. It showed a clear separation between the expression profiles of the first and second peaks (Fig. 2a, 45.9% of the variance between the peaks is retained by the first principle component, PC1). Distinct profile separation was also observed between samples originating from different carbon sources, CB and MCC (Fig. 2a, 25.4% of the variance is retained by the second principle component, PC2). To evaluate the similarity between samples, we quantified the Pearson correlation coefficient [37] for each pairwise combination of sample intensities (Fig. 2b). This showed that proteins in the same molecular-weight fraction (either I or II) have similar protein expression profiles, despite the fact that they were derived from different growth substrates. Upon comparing the number of detected proteins in each peak or substrate, we observed a large overlap between the samples (Fig. 2c). Further comparison of the detected proteins to known CAZymes revealed hundreds of proteins containing cohesins, dockerins and CBM modules, which are detailed in Fig. 2d.

**Fig. 3:**
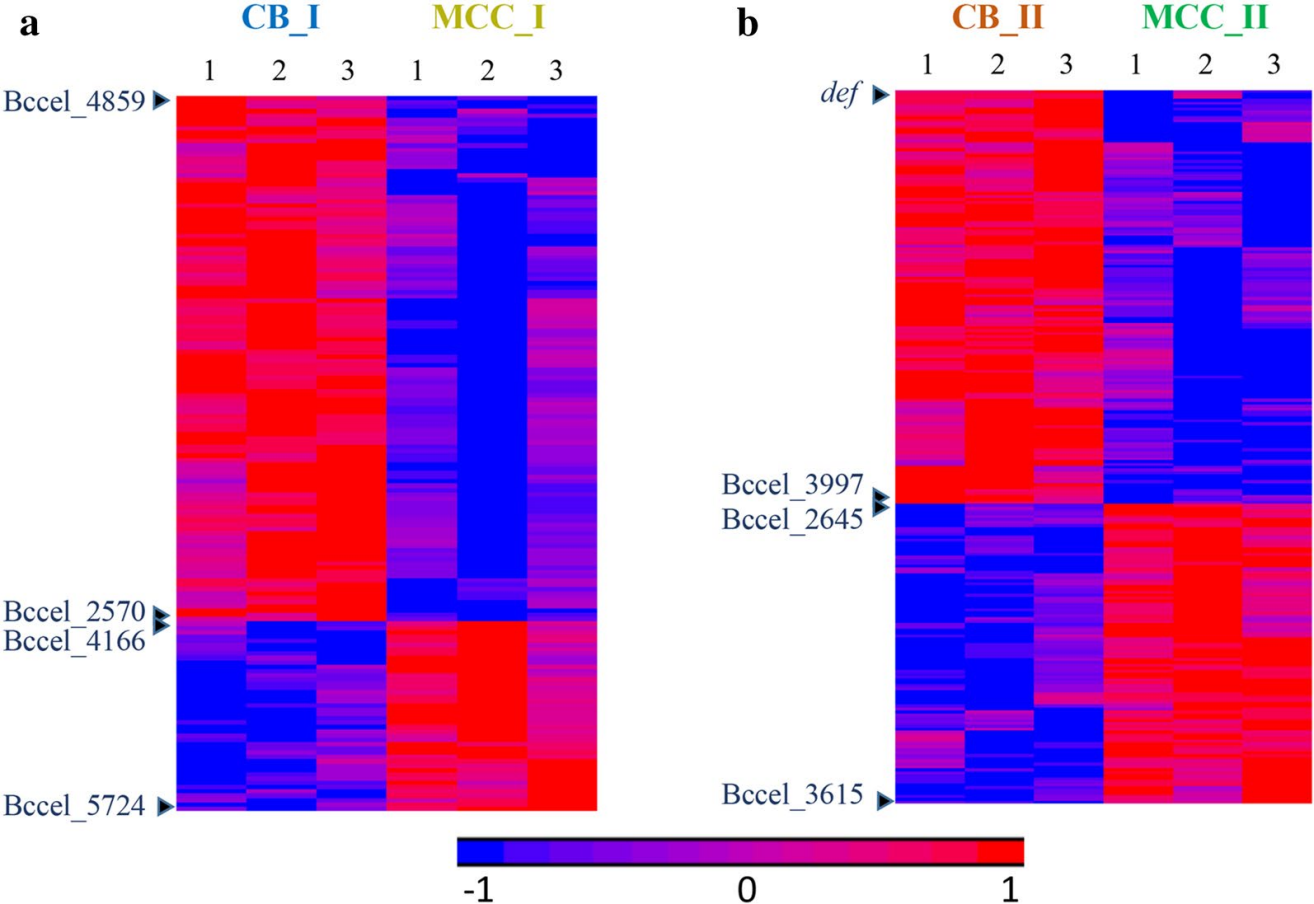
Differentially expressed proteins between carbon sources in the different molecular size fractions. Heatmap of intensities of **a** 166 proteins which showed significantly different intensities between cells grown on CB and MCC in peak I, and **b** 245 proteins which showed significantly different intensities between cells grown on the different substrates in peak II. Heatmaps were generated using LFQ intensities (log2), where zero intensity values were imputed to 10. Rows were standardized, and clustered by Hierarchical clustering using the Euclidian method and average linkage. Differential proteins had |log2 fold change| ≥ 1 and FDR *q*-value ≤ 0.1. Full list of gene names and intensities is detailed in Additional file 3: Table S2. Genes at the top and bottom of the heat maps and at the boundaries between high and low intensity areas are indicated (see Additional file 3: Table S2). Triplicates of two molecular-weight peaks of the two carbon sources (cellobiose—CB and microcrystalline cellulose—MCC) were clustered hierarchically. Numbers from 1 to 3 at top represent the different triplicates from the two substrates

Statistical analysis of protein intensities revealed 166 proteins with significantly different expression between the substrates in peak I (Fig. 3a, Additional file 3: Table S2A), and 245 proteins showed significant difference between CB and MCC in peak II (Fig. 3b, Additional file 3: Table S2B).

### Detection of cellulosomal components

#### Scaffoldins

**Fig. 4:**
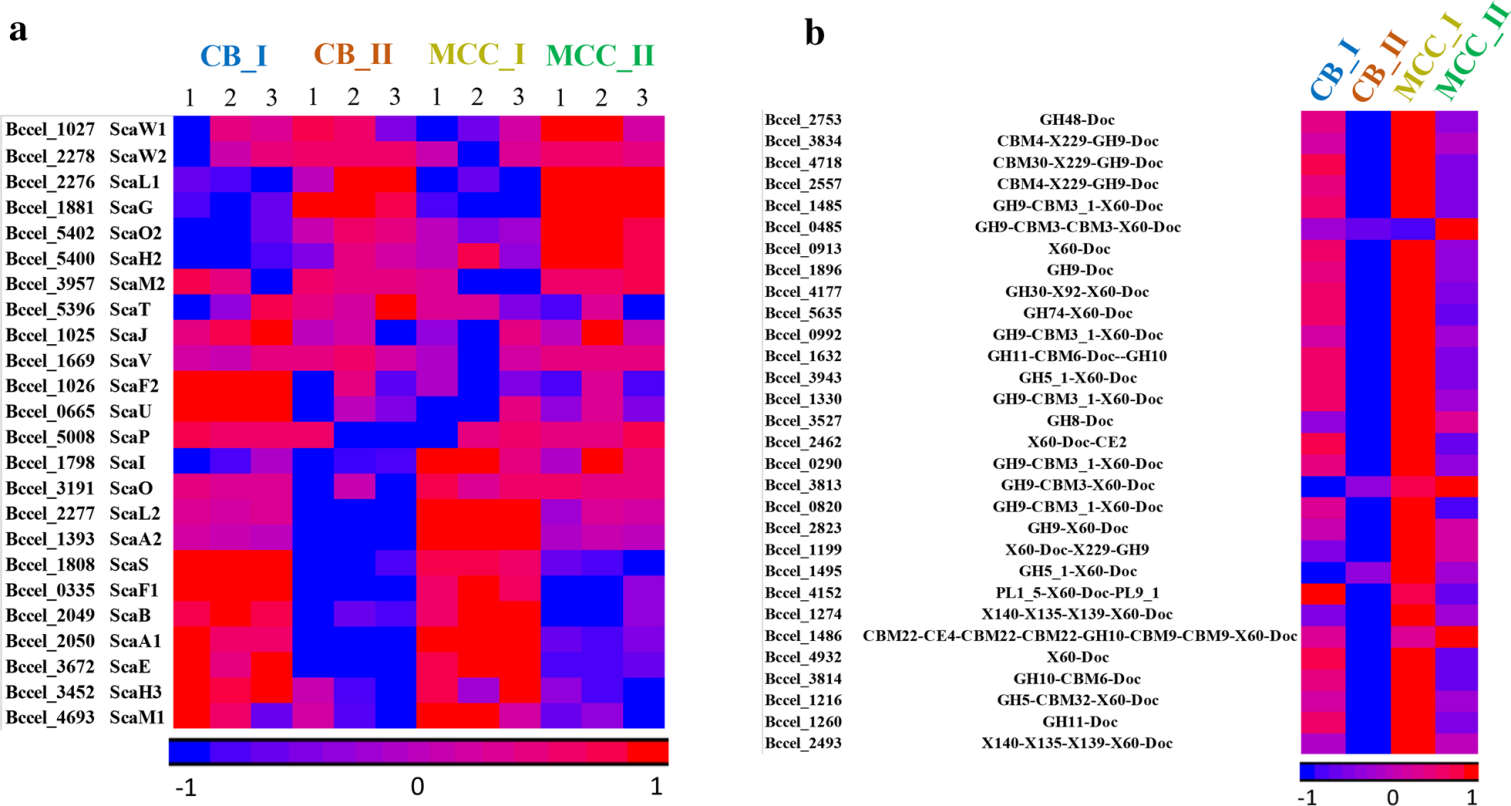
Protein abundance of cellulosomal components, detected in different size fractions (I or II) from supernatant fluids derived from cells grown on soluble and insoluble cellulosic carbon sources (CB or MCC). Heatmap of protein expression values of **a** 24 *B. cellulosolvens* scaffoldins (Additional file 4: Table S3A); **b** 30 (of 166) selected most abundant dockerin-containing proteins (Additional file 4: Table S3B). Names of genes (locus tags) and their CAZy modules are noted. Heatmaps were generated using LFQ intensities (log2), where zero intensity values were imputed to 10. Rows were standardized and clustered by partitional clustering using the Euclidian method. Full list of gene names and intensities is detailed in Additional file 1: Table S1. Numbers from 1 to 3 at top represent different triplicates from the two growth substrates. CB: cellobiose; MCC: microcrystalline cellulose; Doc: dockerin; GH: glycoside hydrolase; CBM: carbohydrate-binding module; CE: carbohydrate esterase; PL: polysaccharide lyases

The *B. cellulosolvens* genome possesses 32 cellulosomal structural scaffoldins. In our previous study, we reported 31 scaffoldins [26], but during the course of MS analysis we identified a new scaffoldin, ScaO2 (Bccel_5402), that was not reported previously. Of the 32 scaffoldins, 24 were identified by proteomic analysis, in addition to significant numbers of cellulosomal enzymes (Fig. 4a; Additional file 4: Table S3A; for modular organization of the detected scaffoldins, see Additional file 5: Figure S2). The major and largest cellulosomal proteins were detected and evaluated by two analysis methods (LFQ and iBAQ), in order to obtain qualitative and quantitative estimation of cellulosomal composition. To follow the discourse below, please refer to Figures 4 and 6 in Zhivin et al. [26].

ScaA1 is a primary scaffoldin that includes 11 type II cohesin modules (that were shown to bind type II dockerins of the various enzymes) [26], a type I dockerin (for binding to type I cohesins of the various anchoring and selected cell-free scaffoldins) and a CBM3. ScaA1 was found to be the second most abundant scaffoldin. In similar cellulosomal systems that were examined, including those of *C. clariflavum* and *C. thermocellum* [30, 38], ScaA (the ScaA1 ortholog) was found to be the most abundant scaffoldin in each case. Probably due to its size and the presence of the CBM3, ScaA1 serves as the most significant enzyme-integrating protein. We therefore normalized the intensities of all cellulosomal proteins to that of ScaA1, in order to facilitate interpretation of the results. This enabled us to estimate the relative fold change of cellulosomal components to the major primary scaffoldin, such that the intensities of ScaA1 for all the iBAQ results would be defined as “1.000”. Similarly, we selected ScaA1 in LFQ CB I to normalize the other values for comparison (Additional file 4: Table S3A) [15, 22].

Intriguingly, the most abundant scaffoldin was found to be ScaE with seven type I cohesins, able to bind type I dockerins of ScaA1, ScaA2, and ScaL2 [26]. ScaE is a cell-free scaffoldin and does not have any additional features except cohesin modules and the intermodular linkers. The combination of ScaE with its seven cohesin, that could potentially bind seven ScaA1 molecules, would create a large cellulosomal complex with 77 enzymes. Interestingly, iBAQ comparison revealed the highest fold change of ScaE in lower-molecular-weight fractions for both CB and MCC (3.5- and 2.1-fold, respectively), while in CB I it was 1.77- and 2.1-fold in MCC I. This means that theoretically we would have a significant portion of ScaE not being occupied, and this would explain its high abundance in the lower-molecular-weight fraction. The complex cellulosomal structure would include additional anchoring scaffoldins, such as ScaB, ScaF1, and ScaF2, that could participate in binding type I dockerin-possessing primary scaffoldins. In our previous study [26], the cell-free ScaE was shown to bind primary scaffoldins ScaA1, ScaA2, and ScaL2 and may thus play an important role in degradation of remote cellulosic substrates. ScaE orthologs were found to be comparatively abundant in *C. thermocellum* and *C. clariflavum* [30, 38].

The ScaF1 anchoring scaffoldin with a single type I cohesin and an SLH module showed comparatively high abundancy levels. It shows some similarity to ScaF2 which showed much lower intensities in all fractions. ScaF1 showed the highest intensity values among the anchoring scaffoldins. Theoretically, it might anchor a single ScaA1, ScaA2, ScaL1, or ScaL2, although the binding assays showed a clear preference for the ScaL2 and ScaR3 dockerins (ScaR3 was not expressed). Therefore, we are able to identify in the supernatant fluids scaffoldins that are presumably cell-bound. ScaF2 showed a lower score, which may indicate its low level of expression or the possibility that the protein stays partly bound to the bacterial cell wall and was not extensively released into the supernatant fraction.

The second largest primary scaffoldin, ScaA2, showed relatively high intensity for MCC I (3.03 times lower than ScaA1) and MCC II (6.25 times lower than ScaA1) but appeared in much lower amounts than ScaA1 in all other fractions. This fact is surprising since ScaA2 is a large scaffoldin with 10 cohesins and a type I dockerin and was shown to have similar binding properties as ScaA1 [26]. Compared to ScaA1, though, it lacks the CBM3, but its cohesin sequences are very close to those of the ScaA1 cohesins.

ScaG showed significant fold change in the lower-molecular-weight fraction in comparison to the high-molecular-weight peaks for cells grown on both substrates. ScaG possesses a single enzyme-binding type II cohesin and a CSBM (cell surface-binding module) that anchors the scaffoldin to the cell surface. As expected, the intensity of ScaG was very low in the higher molecular-weight fractions. In contrast, ScaG was indeed found to be very abundant in fractions CB II and MCC II (2.79 and 1.67-fold higher than ScaA1, respectively). In recent mass spectrometry analysis of the *C. clariflavum* cellulosome [38], the ScaG ortholog was shown to be the only scaffoldin subunit found to be more abundant than ScaA in any of the fractions. An additional ScaG ortholog, OlpC from *C. thermocellum*, was also an abundant protein on the bacterial cell surface [15, 39]. It was suggested [39] that OlpC may serve as a transit station or a shuttle vector for cellulosomal enzymes on their way to creating more complex cellulosomes. OrfXp, another ScaG ortholog in *C. cellulolyticum* [40], was suggested to have similar function. The surface-binding CSBM of ScaG is orthologous to those of the *C. clariflavum* ScaG and *C. thermocellum* OlpC, which are different than the SLH module of the anchoring scaffoldins.

High intensities of ScaH2 in cells grown on both substrates were perhaps surprising. ScaH2 is a small primary scaffoldin possessing one type II cohesin and a type II dockerin. It was found to bind strongly to type II dockerins with a somewhat different preference compared to ScaA1 and ScaA2 [26]. Interestingly, the ScaH2 cohesin bound to the dockerins of several hemicellulases but not to that of the abundant GH48 exoglucanase. ScaH2 may also bind primary scaffoldins that possess a type II dockerin (such as ScaH3, ScaI, and ScaO), which all possess single cohesins.

ScaL2 showed similar results for both cell-growth substrates and chromatographic fractions. It is a primary scaffoldin with three type II cohesins and a type I dockerin. ScaL2 was found to bind strongly to enzyme-borne type II dockerin modules and weakly via its dockerin to the cell-free ScaE or cell-anchoring ScaF1, ScaF2, and ScaU. Despite lower overall abundancy, the fold change of ScaL2 was closer to that of ScaF1.

Interestingly the largest anchoring scaffoldin ScaB showed comparatively low expression values. It appeared more than 30 times lower than ScaA1 in all fractions for both substrates, despite having 11 type I cohesins that would be available for interaction. ScaB cohesins from *B. cellulosolvens* exhibited strong specificity for the dockerins of ScaA1 and ScaA2 [26]. In contrast, previous proteomic studies in *C. clariflavum* revealed that its adaptor scaffoldin ScaB was detected in comparatively high amounts that fits the exact model of occupation by ScaA [38].

#### Dockerin-containing enzymes

**Fig. 5:**
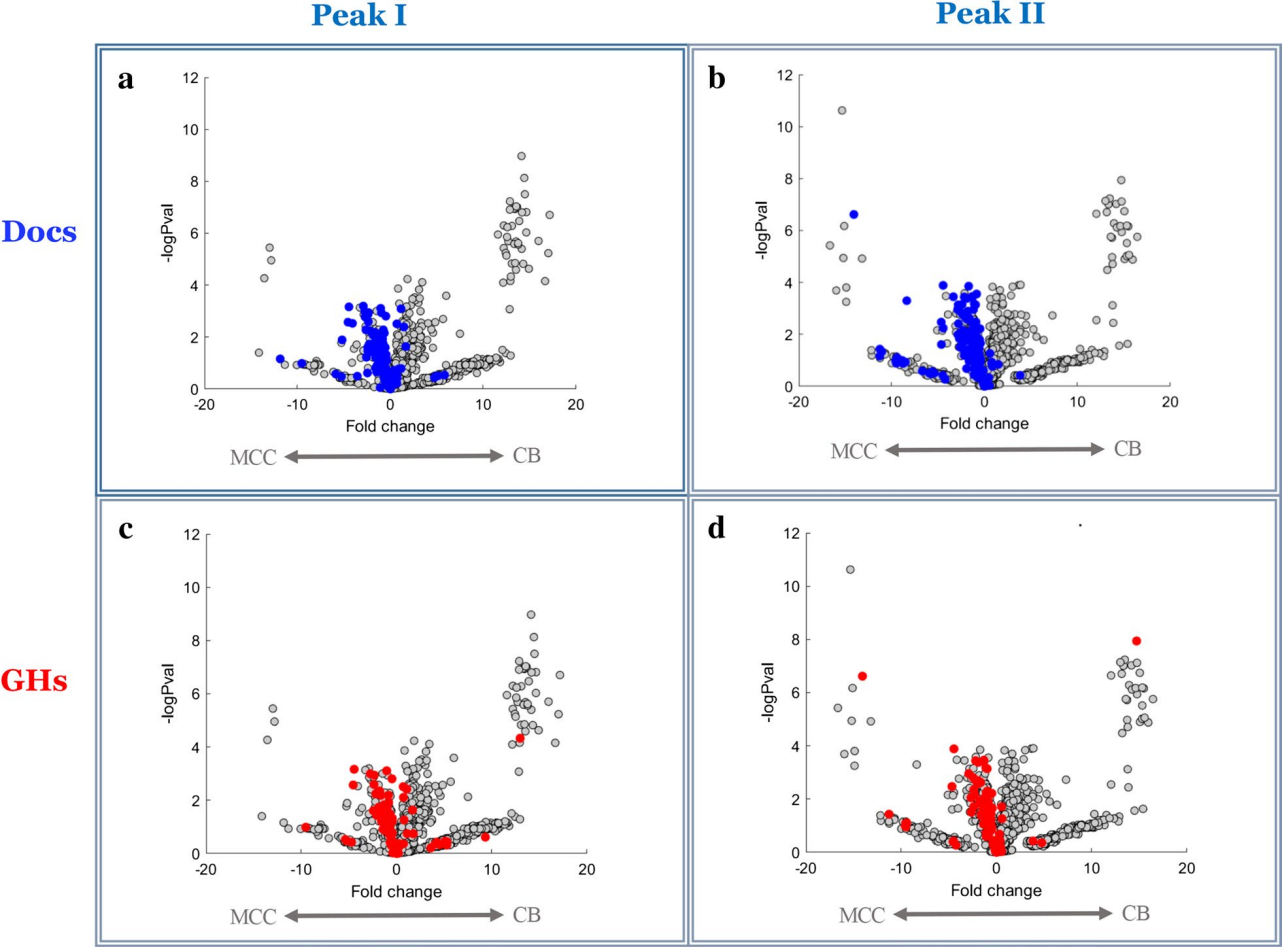
Distinct distribution of GH- and dockerin-containing proteins between the peaks. Volcano plots highlighting differences in abundance of proteins between the growth substrates (either MCC: microcrystalline cellulose; or CB: cellobiose) in the different peaks (I or II). All proteins which were detected in this study appear as gray dots, with the fold change (log2) of their abundance (*x* axis) and the significance level (*y* axis). Colored dots represent proteins containing either dockerin (blue) or GH (red) modules. The graphs indicate higher expression levels on cellulose, compared to cellobiose-containing growth medium

Of the 212 putative dockerin-containing ORFs which are coded in the genome, 166 were detected in this work (Additional file 4: Table S3B, Additional file 6: Figure S3). This is the highest number of cellulosomal catalytic subunits that were found to be expressed in a single cellulosome study. Considering the largest arsenal of enzymes in the *B. cellulosolvens* genome, this result is, perhaps, anticipated. Figure 4b represents the intensities of the 30 most abundant dockerin-possessing proteins, correlated to the growth substrate and molecular-weight fraction. Visualization of the protein intensities on a volcano plots shows that most dockerin-containing proteins were significantly expressed in MCC-rather than in CB-containing medium, for both peaks (Fig. 5a, b). This trend may be explained by higher concentrations of dockerin-possessing enzymes in cells grown on the insoluble cellulosic medium that requires higher degradation capabilities.

We examined the molar ratios of cohesins and dockerins within the peak population, in order to better understand the possible ways of cellulosomal assembly. The molar ratios were calculated by considering the number of vacant type II cohesins on the expressed scaffoldins and the number of expressed dockerin-possessing enzymes (we assumed a molar ratio of 1 for monovalent scaffoldin with type II cohesin and one dockerin subunit). Examination of the molar amounts of dockerin-containing enzymes revealed high compatibility with the vacant type II cohesins in high-molecular-weight fractions for both growth conditions (Table 1). Intriguingly, in the lower-molecular-weight fractions of both substrates, the molar amounts of the enzymes were about twice those of the higher molecular-weight fractions, meaning that there were twice the available enzymes than vacant cohesins, indicating large numbers of excess enzymes that will not associate with cohesins at a specific point. In other cellulosomal models, the enzymes are usually found in significant excess, depending on the substrate used [30, 38, 41, 42].

**Table 1:** Ratios of molar amounts of available type II cohesins versus molar amounts of type II dockerins

	CB I	CB II	MCC I	MCC II
Scaffoldins	12.78	16.94	15.55	17.49
Dockerins	15.30	29.62	16.35	30.16

The molar amounts of scaffoldins were calculated per fraction by summing the iBAQ intensities obtained from multiplying the normalized intensities of type II cohesin-bearing scaffoldins by the number of cohesins on expressed multi-valent scaffoldins. The molar amounts of dockerins were calculated per fraction by summing the normalized intensities of expressed type II dockerin-bearing enzymes

We noticed significant numbers of X-modules/domains (110 out of 166 [Additional file 4: Table S3B]), especially the X60 module, that were associated in tandem with many of the enzyme-bearing dockerins. This fact emphasizes the importance of this module in cellulosome assembly in *B. cellulosolvens* and is unusual for cellulosomal bacteria, owing to the presence of the X-Doc modular dyad described mainly for anchoring scaffoldins and certain GH10-family enzymes, e.g., Clocl_2194 of *C. clariflavum* [38]. Similar to *C. clariflavum*, *A. cellulolyticus*, and *C. thermocellum*, we observed comparatively small numbers of non-cellulosomal enzymes (36 non-cellulosomal compared with 166 cellulosomal). This again highlights the efficiency of the cellulosome system, but also highlights possible complementary interactions of the two cellulase paradigms.

### Glycoside hydrolase representatives

It was reported previously in *C. thermocellum* that the expression levels of exoglucanases and endoglucanases were elevated on cellulose-versus cellobiose-containing growth media [15, 22, 42]. Our findings show similar results (Additional file 7: Table S4 and Fig. 5c, d). Putative endoglucanases, including GH9 (8 enzymes), single GH5 and GH26 families, were highly expressed on cellulose. The second highest expressed enzyme (after the GH48 exoglucanase) is a putative endoglucanase CBM4-X229-GH9-Doc (Bccel_3834), which was upregulated on cellulose-containing medium and mostly abundant in the highest molecular-weight-peak, indicating its significance to the cellulosomal function. The general trend shows increased levels of endoglucanase expression on cellulose, a finding consistent with results obtained by Dror et al. in *C. thermocellum* [43].

The exoglucanases were similarly upregulated on cellulose. Putative *B. cellulosolvens* exoglucanases are represented mostly by GH48 (3 enzymes) and GH9 (12 enzymes). Consistent with previous reports in other cellulosome-producing bacteria [22, 30, 38, 44, 45], the most abundant putative exoglucanase in *B. cellulosolvens* is GH48 (Bccel_2753). The family 48 glycoside hydrolase enzymes represent a major component of all known cellulosomes, as well as some non-cellulosomal bacterial systems [6]. Bccel_2753 shares similarity with GH48 (Clocl_4007) from *C. clariflavum* and exoglucanase Cel48S from *C. thermocellum* [46, 47]. The combination of highly expressed cellulases from families GH48 and GH9 was reported previously as well. In *C. thermocellum* [14, 48] and *C. termitidis* [49], the latter two glycoside hydrolase families include enzymes that were found to be mostly abundant cellulosomal exoglucanases. Interestingly, the previously studied [50] Cel48A exoglucanase (Bccel_0895) was expressed at lower intensity but was still comparatively high and appeared in all fractions. Compared to ScaA1, the quantity of GH48 in each complex is 3- to 7.5-fold higher. Similar to findings in *C. clariflavum* [38], the highest expression levels of GH48 were found in CB II and MCC II, while the GH48:ScaA1 ratio in those fractions was compatible with that in *C. clariflavum* (7.23 for MCC II).

Hemicellulases were relatively abundant among the carbohydrate-degrading enzymes. Multiple putative xylanases were identified, represented by GH10 (11 enzymes), GH11 (6 enzymes), a single GH30 and GH62 (Additional file 7: Table S4). This group included the highest number of multi-functional enzymes—9 out of 15 xylanases. The prevalent CBM families included CBM6, CBM9, and CBM22, all able to bind xylan [51]. Interestingly, the abundance of xylanases was similar between the growth substrates with no significant differences. This could be explained by the absence of hemicellulose in the growth medium, but, as observed for other cellulosome-producing bacteria [43], the bacterium appeared to keep basal expression levels in case of substrate availability. Xylan degradation products could be further degraded by a β-xylosidase represented by GH43 (Bccel_1712).

Additional putative hemicellulases, involved in the hydrolysis of arabinose (GH43, GH53) and mannan (GH2, GH5_8, GH26) were also detected. Clustered GH5_8 cellulosomal enzymes (Bccel_2491, Bccel_2492) were upregulated in cellulose-containing growth medium. Since *B. cellulosolvens* is not able to grow on hemicellulose, the bacterium may apply its hemicellulases to gain access to the preferred cellulosic substrate of the plant cell wall.

Of the 148 GH-containing ORFs found in the genome, 109 GH modules (102 GH-containing ORFs, some representing multi-functional enzymes) were expressed (Table 2; Additional file 7: Table S4). Almost all of the enzymes were expressed under both growth conditions, except CBM2-GH5_1 and GH10-CBM9-CBM9 (Bccel_4191 and Bccel_5603, respectively) that were specific to cellobiose, while GH8-Doc-CE4 and GH3 (Bccel_0446 and Bccel_3298, respectively) were specific to cellulose. Three enzymes (Bccel_1373: GH25, Bccel_3076: GH11-CBM6-Doc-GH10 and Bccel_3093: GH11-GH10-Doc-X124) appeared only in the high-molecular-weight peaks, while Bccel_0905 (GH3) and Bccel_1425 (GH10) appeared only in the low-molecular-weight peaks under both growth conditions.

**Table 2:** GH modules expressed in *B. cellulosolvens*

Glycoside hydrolase	2	3	5	8	9	10	11	13	16	18	23	25	26	27	30	39
Expressed modules	1	6	11	4	38	11	6	1	2	3	–	1	3	–	3	–
Expressed dockerin-containing modules	–	1	8	4	33	7	5	–	2	1	–	–	3	–	3	–
Genome-wide	1	7	11	4	40	15	8	6	4	6	2	1	5	1	3	1

Glycoside hydrolase	43	44	48	51	53	57	62	63	67	74	75	81	94	95	115	Total
Expressed modules	7	1	3	–	1	–	1	–	1	1	–	1	2	–	1	109
Expressed dockerin-containing modules	6	1	3	–	–	–	1	–	–	1	–	1	–	–	–	80
Genome-wide	11	2	3	1	1	1	1	1	2	2	2	1	3	1	1	148

GH9 is the largest enzyme family in *B. cellulosolvens* represented by 40 enzymes, and 33 of them possess a dockerin. We found 38 expressed GH9 representatives, and all 33 cellulosomal GH9 enzymes were expressed. Most of the enzymes, possess a CBM and/or X-modules/domains, in addition to the GH9 and the dockerin. GH9 enzymes are common in cellulosomes of *C. clariflavum* [52] and *C. thermocellum* [53] and other species [54–56]. The most expressed GH9 enzyme in *B. cellulosolvens* is Bccel_3834 (CBM4-X229-GH9-Doc). Its intensities were about twofold lower than those of the most abundant GH48 cellulosomal enzyme (Bccel_2753). Bccel_3834 is annotated as an endoglucanase and shares high similarity with Cel9K from *C. thermocellum* (recently determined to be an exoglucanase [57]) and Clocl_3917 from *C. clariflavum*. In general, the levels of GH9 enzymes were higher in MCC-derived cellulosomes but not as significant as those in *C. clariflavum* and *C. thermocellum* [30, 38]. This is consistent with previous findings in *C. thermocellum*, which showed an increase in GH9 endoglucanase expression during cultivation on insoluble cellulose-containing growth media [15, 22, 43].

The next most abundant GH family is GH5 with all of the 11 GH5-containing genes in the genome expressed, suggesting that all bacterial GH5 enzymes participate in carbohydrate degradation. Most of the enzymes were found in both CB- and MCC-derived cellulosomes, and only Bccel_4191 (CBM2-GH5_1) was missing in the MCC-grown cultures and second peak of CB-grown media, while its level in CB I was comparatively low. GH5 enzymes represent a wide range of enzymatic activities (notably cellulase, xylanase, and mannanase activities), and sequence examination indicates that those of *B. cellulosolvens* are probably endoglucanases.

The presence of expressed enzymes from the GH10 family was also relatively high: 11 enzymes (out of 15 genome-wide GH10s), while six of the expressed enzymes were cellulosomal and one possessed an SLH module (Bccel_1491, CBM22-CE4-CBM22-GH10-CBM9-CBM9-SLH-SLH-SLH), signifying its attachment to the bacterial cell surface. This multi-modular enzyme, that showed the highest intensity among GH10 family, is a distinctive xylanase. Similar enzymes are highly expressed in other cellulosome-producing bacteria [38]. Its SLH module anchors the enzyme to the cell wall, while two different types of CBM presumably target the enzymatic modules to the preferred substrate (xylan). A second expressed GH10 enzyme possesses a similar structure, but the main difference is the substitution of the SLH module by a X60-dockerin modular dyad and an additional CBM22 which purportedly binds xylan. The structural elements of both enzymes suggest that they function as endo-xylanases. Generally, we find a relatively large number of multi-modular enzymes in *B. cellulosolvens*: 8 out of the 11 expressed GH10-containing enzymes are multi-functional.

GH11 family xylanases also showed a significant presence. Six out of eight enzymes were expressed, some of which overlapped with the GH10 enzymes as multi-functional enzymes, since in four cases (Bccel_1632, Bccel_3733, Bccel_3076 and Bccel_3093) a second module included a GH10 in addition to the GH11 module. In this family, the highest intensity was shown by the bi-functional cellulosomal enzyme Bccel_1632 (GH11-CBM6-Doc-GH10). Similar to the GH10 group, the members of this group of enzymes are also characterized as putative endo-xylanases.

GH13 is represented by a single expressed non-cellulosomal enzyme (Bccel_2759) from sub-family 9 (X104-CBM48-GH13_9). The enzyme appeared in both substrates with low intensity values. This putative 1,4-alpha-glucan branching enzyme (amylase) includes an interesting CBM48, annotated as a glycogen-binding function, which is characteristically appended to GH13 modules.

Two cellulosomal GH16 enzymes were detected. The highest intensity was shown by the bi-functional GH43-CBM13-Doc1-GH16 (Bccel_1738). An additional GH16 enzyme includes a CBM4.

The GH18 family was represented by two enzymes. A non-cellulosomal protein included a CBM50 which was shown to be attached to different GH families [51] including GH18. Another GH18 enzyme is cellulosomal and possesses a X60-Doc modular dyad. The GH18s exhibit a variety of activities, including chitinase and lysozyme-like activities.

A single non-cellulosomal GH25, annotated as a putative lysozyme was expressed at low levels and is unique to the high-molecular-weight fraction.

Three cellulosomal GH26-containing proteins were also detected. Two of them included CBM35, representing putative mannosidase function.

Three cellulosomal GH30 putative xylanases were expressed. The enzyme that showed lower intensity (Bccel_5541) was examined experimentally and was shown to bind strongly to a range of primary scaffoldin-based cohesins [26]. Two GH30 xylanases were highly expressed in *C. clariflavum*, but not in *C. thermocellum* [38].

Seven of eleven GH43 proteins were expressed, six of which possess a dockerin module. A highly expressed bi-functional cellulosomal enzyme (Bccel_1738, GH43-CBM13-Doc-GH16) probably functions as a xylanase or xyloglucanase.

The GH94 family was represented by two putative carbohydrate phosphorylase enzymes. Seven additional GH families were represented by a single expressed enzyme: GH44, GH53, GH62, GH67, GH74, GH81, and GH115, suggesting additional xyloglucanase, arabinofuranosidase, galactanase, endo-β-1,3-glucanase, and/or glucuronidase activities. All of the latter families are commonly found as components of cellulosomes.

In total 15 multi-functional cellulosomal enzymes with more than one catalytic module were expressed out of 17 found in the genome. Multi-functional enzymes were described previously and are common in cellulolytic and cellulosome-producing bacteria [38, 58–60].

In addition to GH catalytic enzymes, 20 CE-containing enzymes (7 of which included a GH module) and 5 PL-containing enzymes were expressed at varying expression levels.

### Clustered catalytic ORFs

**Fig. 6:**
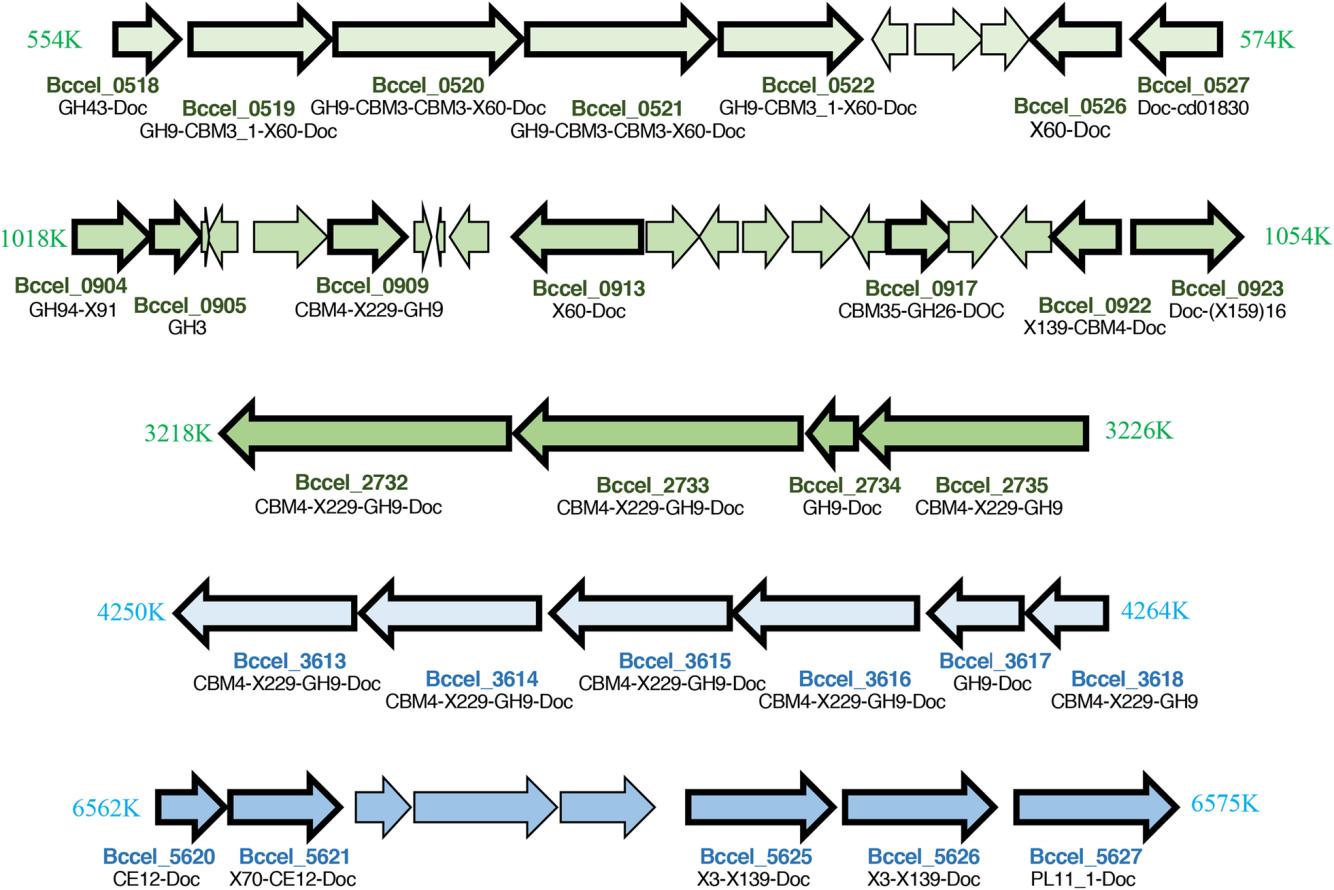
Clustered organization of GH-containing genes expressed and detected in this study. The genomic environment of selected clusters of CAZyme-coding genes is presented. Expression values are detailed in Additional file 7: Table S4

The genomic location of adjacent ORFs were examined in order to reveal clusters of expressed cellulosomal and free enzymes and to try to follow the clustering of functional groups. Interestingly, some of the enzymes are clustered on the genome according to the GH family type, and some are expressed with similar intensity values (Fig. 6, Additional file 8: Table S5), raising the possibility of operon structure and common regulation, due to similar function and expression levels. The genes encoding the enzymes are scattered along the genome, mostly in small “islands” with or without gaps of one or a few ORFs. Enzymatic gene clusters were reported in additional cellulosome-producing mesophilic bacteria, including *Clostridium termitidis*, *C. cellulolyticum*, *C. josui*, *Clostridium cellulovorans*, and *Clostridium acetobutylicum* [42, 61, 62] as well as thermophilic anaerobes—*C. thermocellum* [63] and the non-cellulosomal, cellulolytic *Caldicellulosiruptor bescii* [64].

The most noteworthy expressed cluster (Bccel_0518-22; Bccel_0526-27, Fig. 6) includes seven ORFs, all cellulosomal. Four of them include GH9 and CBM3c’s, one enzyme with a GH43, a single dockerin-containing ORF and a putative SGNH_hydrolase (putative esterase or lipase [65]). This group of genes (Bccel_0518-22; Bccel_0526-27) encodes cellulosomal cellulases with similar architecture and most probably similar complementary functions. The GH9 modules here are associated with single or double CBM3s associated with cellulose-binding and, in some cases, associated with processive endoglucanase activity.

A group of six enzymes (Bccel_3613-18) also represents an interesting cluster of GH9 cellulases. Four of the six possess the same modular structure (CBM4-X229-GH9-Doc); one of them (Bccel_3618) lacks a dockerin and is therefore non-cellulosomal, and Bccel_3617 lacks a CBM4. The first four enzymes of the cluster have the same modular architecture as two of the most highly expressed enzymes (Bccel_3834 and Bccel_2557), but their expression levels are much lower.

An additional four putative GH9 cellulases, are clustered together on the genome (Bccel_2732-Bccel_2735). Two of them share a similar structure (CBM4-X229-GH9-Doc, again like the two highly expressed GH9 enzymes), while the third enzyme has no dockerin and the fourth ORF has only GH9-Doc.

An additional notable cluster of CAZymes includes genes from GH94, GH3, GH9 and GH26 families (Bccel_0904 to Bccel_0923, not sequentially). This cluster is characterized by a comparatively large number of CBMs: CBM4, CBM9, and CBM35.

### Catalytic activity of the cellulosomal fractions

**Fig. 7:**
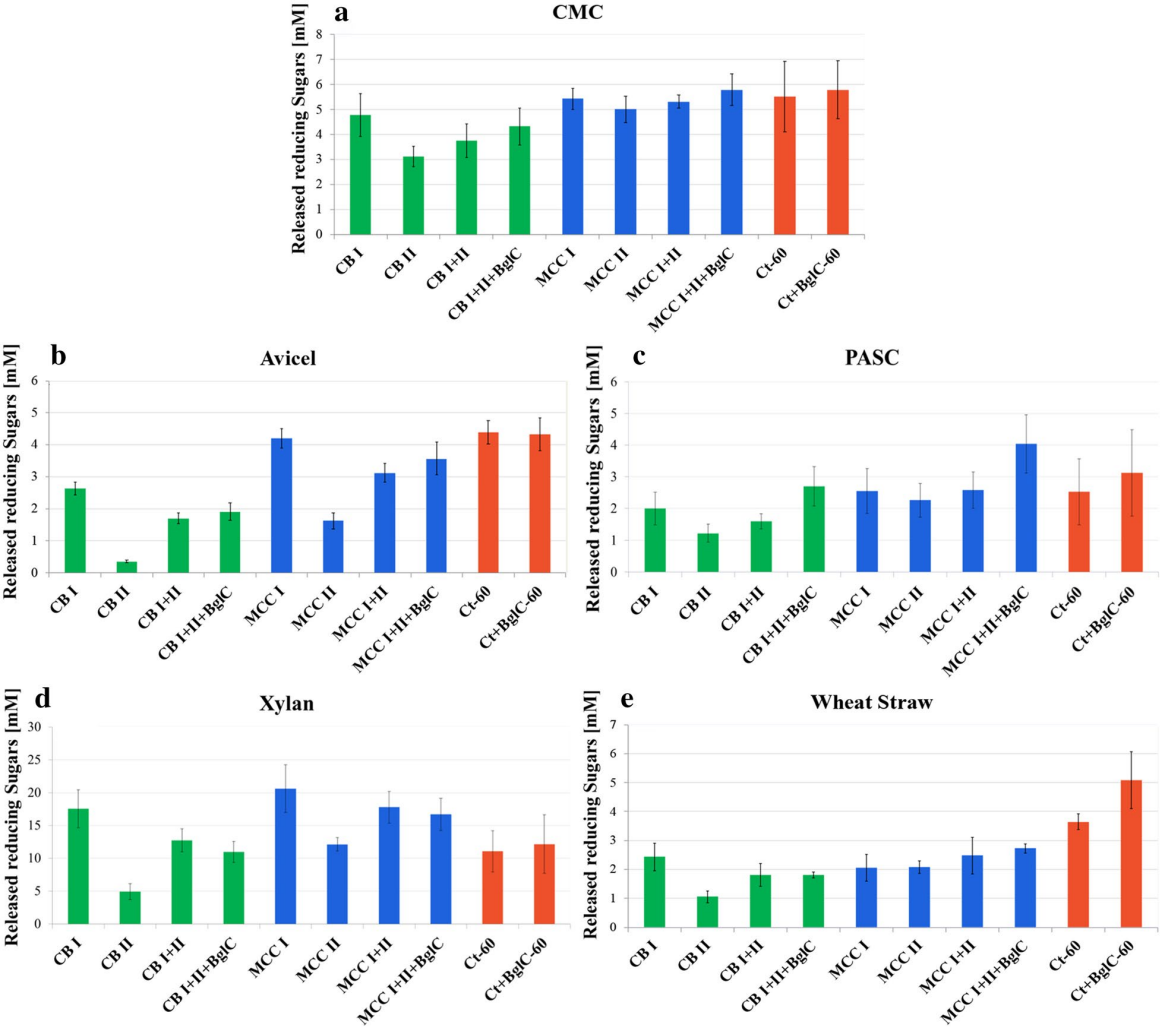
Hydrolysis of various carbohydrate substrates by cellulosome fractions of *B. cellulosolvens*. Two fractions (I: high-molecular-weight, and II: lower molecular-weight, separated by gel filtration chromatography as described in the “Methods” section), containing cellulosomal complexes derived from cells grown on either cellobiose (CB) or microcrystalline cellulose (MCC), were examined for catalytic activity on **a** CMC (carboxymethyl cellulose), **b** MCC (Avicel), **c** PASC (phosphoric acid-swollen cellulose), **d** beechwood xylan, and **e** wheat straw, in order to demonstrate their degradation abilities. The cellulosomal fractions were tested (at 40 °C, optimal activity temperature) separately or combined (combination of peak I and II from the same growth medium), in order to examine possible synergistic effects. In order to avoid possible inhibition by degradation products, recombinant β-glucosidase from *Thermobifida fusca* (BglC) was added to the catalytic reactions of the combined fractions of *B. cellulosolvens* and to the *C. thermocellum* cellulosome. BglC was chosen due to its optimal temperature (50 °C), and it was active both at 40 °C for *B. cellulosolvens* activity and at 60 °C for *C. thermocellum* (Ct) activity. The *C. thermocellum* cellulosome (cells grown on MCC as substrate) was tested as a positive control for catalytic activity of the *B. cellulosolvens* cellulosomes

The catalytic activities of the isolated cellulosomal fractions were examined on five substrates: CMC (carboxymethyl cellulose), Avicel (microcrystalline cellulose), PASC (phosphoric acid-swollen cellulose), beechwood xylan, and pretreated wheat straw. Protein concentration in all samples was 50 µg/ml (Fig. 7). We examined the catalytic activity of the separate cellulosomal fractions (I and II) but also combined the two in order to restore the full cellulosomal function and to test for a probable synergistic effect. For most of the substrates the combination of both fractions I and II yielded activity levels that were higher than those of fraction II alone but failed to reach the activity levels of fraction I, indicating minor or no synergistic effect compared to the separated complexes. In addition, we used recombinant β-glucosidase BglC (WP_011291384.1) from the cellulolytic bacterium, *T. fusca* [31]. Addition of β-glucosidase was shown previously to enhance catalytic activity, due to the elimination of possible enzyme inhibition by cellobiose, the main degradation product [66–68]. The reason we chose this specific β-glucosidase is its optimal activity temperature. Since the optimum of *B. cellulosolvens* activity is 40 °C (data not shown), we wanted to use a β-glucosidase with similar temperature range. The optimal temperature of BglC is 50 °C [31] with a much broader temperature range, and the enzyme is more appropriate for our assay system (40–60 °C). The cellulosome of *C. thermocellum*, known to be a particularly efficient catalytic degrader [7], served as a reference (the tests for this thermostable system were conducted at 60 °C).

Overall, the results depended on the molecular weight of the tested cellulosomal fraction and the growth medium. The cellulosomes derived from cellulose-containing growth medium showed the highest activity results in all fractions, even though for wheat straw degradation there was almost no difference between cellulose- and cellobiose-derived cellulosomes. These results were compatible with the recent findings in *C. clariflavum* [38] and indicated that the activity and cellulosomal content is affected by the growth medium. Mass spectrometry identification did not show significant differences in enzymatic content between the carbon sources, but the differences in intensities were more distinguishable. In general, fractions CB I and MCC I showed high activity, although the results varied depending on the carbohydrate substrate (Fig. 7). This fact emphasizes the efficiency of the cellulosomes, because the high-molecular-weight fractions contain large active cellulosomal complexes, while in lower-molecular-weight fractions smaller complexes and uncomplexed subunits are more abundant. For CMC degradation, MCC-derived cellulosomes showed the highest levels of activity that were compatible with those of the positive control (i.e., *C. thermocellum* cellulosomes). Interestingly all of the MCC fractions showed similar results, whereas, among the CB cellulosome fractions, CB I exhibited the highest level of activity (Fig. 7a). For Avicel as substrate (Fig. 7b), degradation by MCC I was the highest and showed similar results to these of the control. The combination of MCC I and MCC II with addition of BglC showed the second highest levels of Avicel degradation, while MCC II alone was lower than CB combinations except CB II that showed the lowest result. This finding is compatible with the assumption that cellulosomes isolated from cellulose-grown bacteria would degrade microcrystalline cellulose better than cellobiose-derived cellulosomes.

In all tested substrates, CB II consistently showed the lowest levels of carbohydrate substrate degradation, and this is compatible with the mass spectrometry results, which presented the lowest expression values for enzymes in CB II. The third cellulosic substrate examined in our studies was PASC (Fig. 7c). Interestingly, BglC elevated the activity for both CB- and MCC-derived cellulosomes. The combined *B. cellulosolvens* MCC-derived cellulosomal fractions showed similar results on PASC, but the highest level of degradation was achieved by a combination of MCC I, MCC II, and BglC, which was even higher than those of the *C. thermocellum* positive controls. For xylan degradation, the MCC I fraction showed the highest activity (Fig. 7d), which was not affected by the addition of BglC. The activity of the purified *C. thermocellum* cellulosome was comparatively low on xylan and was equivalent to those of the MCC II fraction and the combined CB I and CB II fractions. The combination of MCC I and MCC II showed similar results to CB I, which was slightly higher than that of the *C. thermocellum* cellulosome. Xylan degradation seems to be preferred by *B. cellulosolvens*. This preference can be explained by the high content of xylanases in this bacterium, especially in higher molecular-weight fractions for both substrates, indicating that the xylanases are mostly cellulosomal. Surprisingly, *C. clariflavum* also showed different results for xylan degradation, where, in contrast to *B. cellulosolvens,* the lower-molecular-weight fraction was more active on this substrate than the higher molecular-weight fraction [38]. For wheat straw degradation, all fractions except CB II showed similar results (Fig. 7e). The activity on this natural substrate was much lower than for other substrates. On the natural substrate, the *C. thermocellum* cellulosomes were the most active, especially in combination with BglC.

Addition of BglC to the reaction mixtures slightly enhanced cellulose degradation for all of the combined cellulosome fractions tested and for the *C. thermocellum* cellulosome. For *C. clariflavum*, the addition of BglA (the β-glucosidase from *C. thermocellum*) enhanced the activity for most of the tested substrates except CMC. The elevated activity was particularly apparent for the natural switchgrass substrate [38]. For wheat straw degradation by *B. cellulosolvens* cellulosomes, no significant effect was observed after addition of *T. fusca* BglC, as opposed to the activity of *C. thermocellum*, which was elevated. Apart from the addition of recombinant BglC, the endogenous *B. cellulosolvens* β-glucosidase enzymes might also assist cellobiose cleavage: 5 putative β-glucosidase enzymes from family GH3 were indeed detected in the analysis of the *B. cellulosolvens* proteome (Four free GH3s: Bccel_5320, Bccel_4126, Bccel_3298, Bccel_4484 and a single dockerin-bearing enzyme: GH3-X60-Doc [Bccel_4009], the latter of which may be a part of the cellulosomal complex). Therefore, external BglC may not have affected cellulosomal activity, owing to the presence of endogenous *B. cellulosolvens* β-glucosidases in the fractions. The putative β-glucosidase enzymes expressed in *B. cellulosolvens* show some sequence similarity to known β-glucosidases. It is intriguing that the five putative endogenous β-glucosidase enzymes were all expressed, even though their expression levels were not very high. As stated before [69], cellobiose might inhibit cellulosome degradation activity, and its cleavage to non-inhibitory glucose must be carefully controlled in the cell by β-glucosidases.

## Discussion

Little is known about the unique cellulosome-producing bacterium *B. cellulosolvens.* The aim of this study was to shed light on the intriguing mechanism of carbohydrate degradation in this bacterium. This bacterium bears a substantial pool of carbohydrate-deconstructing enzymes that could be used in the production of biofuels and more generally as tools in the field of biotechnology. This study contributes to cellulosomal research by identifying the most active and important cellulosomal enzymes which possess a type II dockerin. This unique characteristic makes these enzymes particularly interesting, because the majority of the enzymes described in the literature possess type I dockerins.

The proteomic profile of *B. cellulosolvens*, achieved in the present work, supports previous bioinformatic findings [26] and revealed the largest number of cellulosomal proteins expressed in a single bacterium. This provides *B. cellulosolvens* with the potential to assemble an extensive cellulosomal system for efficient plant cell wall degradation. The binding tests that were performed previously [26] provided a clue regarding the possible variant types of cellulosome composition in this bacterium, and with the assistance of protein profiling, we could confirm the actual expressed cellulosomal components and estimate their relative stoichiometry. The multiple expressed catalytic and non-catalytic cellulosomal subunits draw a complicated scheme of cell-free and cell-bound cellulosomal complexes.

In order to describe the ratios of cellulosomal proteins and propose the nature of the possible complexes, we normalized the iBAQ values according to the value of the major primary scaffoldin, ScaA1, in each sample. Normalization versus ScaA1 enabled us to calculate the ratios among the different cellulosomal components within the same sample, both with respect to the amount of ScaA1 versus those of the other scaffoldins, as well as its amount versus those of the various cellulosomal enzymes. Following this calculation, we presumed the observed degradation represented the prevalence of cell-free rather than cell-bound cellulosome, especially in the low-molecular-weight fraction, where ScaE was particularly high. In the high-molecular-weight fraction, ScaE was also the most abundant scaffoldin emphasizing the overall importance of cell-free cellulosomes in carbohydrate degradation for both cellulose- and cellobiose-grown cells (Fig. 4). The fully occupied ScaE would be expected to appear in the high-molecular-weight fraction. In contrast to iBAQ analysis, the LFQ method indicates the importance of the specific proteins according to their intensity values among various samples and enables us to compare the results between the different samples (molecular-weight peaks and substrates).

High expression levels of ScaF1 indicate that cell-bound complexes are important as well. Monovalent ScaF1 can bind a single ScaL2 with three enzymes. As opposed to ScaF1, we would expect to find ScaD (anchoring scaffoldin with three type I cohesins) that theoretically would be three times more effective than ScaF1 or ScaF2. Interestingly, ScaD was not expressed in *C. clariflavum* as well [38].

Most of the protein intensities were higher when the bacterium was grown on cellulose (including ScaA1 and ScaA2), but in the case of ScaA2 the difference is even larger, and a significant fold change was evident in iBAQ data only for cellulose. Evidently, cellulose degradation during bacterial growth requires more resources and the scaffoldins together with its enzymes are thus recruited to this purpose. High expression of the additional large primary scaffoldin ScaA2 in cellulose-derived cellulosomes highlights the need of the cellulosomal machinery for additional catalytic subunits. The salient question here is why would the CBM3-lacking ScaA2 be necessary in the first place? Perhaps, in cellulose-grown cultures, it is necessary to have a dilution of the CBM in the elaborate cellulosome structures in which 11 primary scaffoldins would be incorporated in the anchoring ScaB.

Primary and phylogenetically close ScaL2 and ScaH2 scaffoldins were significantly expressed in comparison to ScaA1 (i.e., > 10%). Similar to ScaA1 and ScaA2, ScaL2 possesses a type I dockerin that could be bound to the cell-free ScaE or to various anchoring scaffoldins. Consequently, it is reasonable that it appeared in relatively high quantities in the high-molecular-weight fractions. The ScaL2 cohesins are phylogenetically relatively distant from those of ScaA1 [26] and could perhaps serve some additional or complementary binding function. The same would be valid for ScaH2, due to the similarity of its cohesin to those of ScaL2. However, ScaH2 mainly appeared in the low-molecular-weight fractions. Likewise, ScaL1 was also prevalent in the low-molecular-weight fractions for both substrates. Interestingly, the type I dockerin of both ScaH2 and ScaL1 showed lower binding activity as opposed to the ScaL2 dockerin, and this could be the reason for this difference in distribution. ScaH2 and ScaL1 would more likely be disconnected from the complexes and thus appear in lower-molecular-weight fractions.

Surprisingly, one complex that is less prevalent is the major anchoring scaffoldin ScaB, which contains 10 type I cohesins that would interact with the enzyme-integrating scaffoldins, such as ScaA1 or ScaA2. This combination would theoretically yield massive complexes of up to 110 enzymatic subunits. The similar levels of partitioning of ScaB between the high- and low-molecular-weight fractions would indicate that many of the ScaB cohesins are unoccupied by ScaA1. We would have expected this complex to be prevalent as in other systems, particularly in *C. thermocellum* [15, 30], but similar to the observations for the major *C. clariflavum* anchoring scaffoldin (ScaC) [38], *B. cellulosolvens* ScaB showed comparatively low abundance.

The variety and high expression levels of some monovalent scaffoldins also indicate their significant function to overall cellulosome function in *B. cellulosolvens*. The proteomic analysis revealed 15 (out of 21 genome-wide) expressed monovalent scaffoldins. In *C. thermocellum*, 4 of the 8 scaffoldins are monovalent (all anchoring), in *C. clariflavum* 4 of the 13 scaffoldins are monovalent (3 anchoring), and in *A. cellulolyticus* 8 of the 16 (3 anchoring, 4 adaptor, 1 free) [52, 55, 70]. The various *Ruminococcus flavefaciens* strains all have a wealth of monovalent scaffoldins [56]. It is thus interesting to consider their possible role(s) in the cellulosomal complex. In the cellulosome of *C. clariflavum*, the orthologous monovalent scaffoldins ScaF and ScaG played a significant role in cellulosome activity [38]. The importance of ScaF as an anchoring scaffoldin is to anchor primary scaffoldins to the bacterial cell. ScaG also is bound to the cell and may bind enzyme-bearing subunits. Another suggestion is that ScaG could serve as a receptor of newly secreted dockerin-possessing enzymes and warehouse function by transiently retaining cellulosomal enzymes at the cell surface before they are assembled onto target multi-enzyme complexes [39]. ScaH2 may serve as a molecular shuttle vector for their transformation to distant complexes [71].

An impressive number of 166 dockerin-containing enzymes (Additional file 4: Table S3B) was revealed by mass spectrometry data. Similar to the scaffoldins, the major differences among the samples reflected expression levels rather than enzymatic composition. The highest expression values for enzymes were obtained in the high-molecular-weight fractions MCC I, followed by CB I. As in *C. clariflavum*, the enzymatic content of CB II and MCC II represented higher ScaA1-to-enzyme ratios. Despite the higher ratio of enzymes to primary scaffoldins in the lower-molecular-weight fractions, the enzyme expression levels in these fractions were lower as well as carbohydrate degradation activity.

MCC I and CB I represented similar molar ratios of type II cohesins to enzymes, representing 0.95 and 0.84, respectively (Table 1). Both ratios are close to “1”, meaning almost absolute compatibility between cellulosomal enzymatic content and available primary cohesins. The compatibility in fraction I could be explained by expressing more or less exact amounts of enzymes to occupy the vacant cohesins in the cellulosome complex, while saving cell energy by not producing large excesses of dockerin-containing enzymes. Despite the equimolar match, we still see a small excess of enzymes, suggesting possible turnover of the enzymes or natural loss of enzymes not reaching the complex. The high presence of the free enzymes in the fraction II indicating that free uncomplexed enzymes or enzymes complexed to small (e.g., monovalent) scaffoldins may be prevalent in low-molecular-weight fractions, whereas cellulosome-anchored enzymes would be found in the high-molecular-weight fractions.

The expression levels of enzymes were also reflected in the activity tests (Fig. 7). MCC-derived cellulosomes showed the highest activity results, while MCC I was the most active fraction. CB II showed lowest results. This leads us to conclude that not only the identity of the enzymes is important for the activity but mostly their expression levels. Moreover, the high-molecular-weight fractions contain large cellulosomal complexes, which are responsible for high activity results, while low-molecular-weight fractions contain smaller cellulosomal complexes and uncomplexed cellulosomal subunits, which would emphasize cellulosome efficiency. The bacterium showed endo- and exoglucanase activities on various substrates during the catalytic activity tests. *B. cellulosolvens* utilizes cellobiose and is not able to grow on some of the degradation products, but those catalytic activities are important to obtain preferred cellulose-derived carbohydrates, while the unutilized sugar polymers may serve other bacteria [72].

## Conclusions

The current study describes the in vivo action of the exquisitely intricate cellulosomal machinery of *B. cellulosolvens* and contributes to the general knowledge of cellulosomes and their involvement in carbohydrate degradation by this bacterium. In this work, *B. cellulosolvens* was grown solely on the two substrates—cellulose and cellobiose—on which it is capable of growing in a reproducible way. Compared to other cellulosome-producing bacteria, e.g., *C. thermocellum* and *C. clariflavum*, growth of *B. cellulosolvens* on natural substrates proved more challenging. In this context, extensive efforts were invested in trying to grow *B. cellulosolvens* on complicated cellulosic substrates, such as wheat straw, but the attempts were largely unsuccessful and, within the framework of the present work, abandoned.

The data obtained in this research revealed both a range of substrates that may be degraded by *B. cellulosolvens* and their degradation products that may serve for future cellulosome research towards biofuel production. We described a multiplicity of elaborate cell-free and cell-associated cellulosomal arrangements in *B. cellulosolvens*. These cellulosomal complexes could be targeted to plant cell wall polysaccharide substrates and include an extremely large diversity of polysaccharide-degrading enzymes which are integrated into the complexes via multiple-scaffoldin assemblies.

One of the main reasons for investigating this fascinating cellulosomal system was to explore its subpopulations for discovery of highly expressed and efficient key carbohydrate-degrading enzymes. More importantly, we tried to understand the relationship between the enzymes and their synergistic effect(s), in order to strive for superior activity results by designer cellulosome technology or cellulosomal cocktails.

The current work analyzes the capacity of the cellulosome-producing bacterium *B. cellulosolvens* to degrade carbohydrates with its extensive machinery of cellulolytic enzymes that has been shown for the first time to be expressed in vivo. The accumulated knowledge of its numerous cellulosomal components enables comparative evaluation of the variety of possible cellulosome architectures and/or cohesin-dockerin functions in the newly characterized, cellulosome-producing bacterium. Moreover, the robust *B. cellulosolvens* cellulosomal system bears potential to provide a significant reservoir of novel components for subsequent cellulosome research, thus promoting future application of designer cellulosomes [73–75] and other types of biotechnological assemblies.

## Additional files

**Additional file 1: Table S1.** Label-free LC-MS/MS raw data analysis of cellulosomal fractions. Raw data from MaxQuant analysis of protein intensities before the normalization step. Data were annotated according to the *Pseudobacteroides cellulosolvens* ATCC 35603 DSM 2993 sequences in UniprotKB.

**Additional file 2: Figure S1.** Chromatographic profile of cellulosomal high-molecular-weight fractions. Gel filtration of supernatant fluids from *B. cellulosolvens* cells grown on two carbon sources: A, cellobiose and B, microcrystalline cellulose. After concentration, the supernatant fluids were loaded onto a Superose 6 Increase gel filtration column. Two major peaks were obtained during the gel filtration process for both substrates. Examination of the peaks revealed two different populations of high-molecular-weight protein complexes that were active on CMC. The column was calibrated using blue dextran and thyroglobulin.

**Additional file 3: Table S2.** Differentially expressed proteins represented in Fig. 3. A, 166 proteins which showed significantly different intensities between substrates in peak I. B, 245 proteins which showed significantly different intensities between CB and MCC in peak II. LFQ intensities (log2) are shown, where zero intensity values were imputed to 10. Differential proteins were selected with cutoffs of |log2 fold change| ≥1 and FDR q-value ≤0.1. CD, cellobiose; Cl, microcrystalline cellulose.

**Additional file 4: Table S3.** Mass spectrometry analysis of normalized cellulosomal proteins. The intensities of all the proteins in four cellulosomal fractions (CB I, CB II, MCC I and MCC II) were estimated by iBAQ and LFQ methods, in order to evaluate their quantity and abundance. The iBAQ intensities were normalized by dividing each value by that of the major primary scaffoldin ScaA1 in each sample, in order to facilitate interpretation of the results and relate the cellulosomal proteins to the major primary scaffoldin within the samples. The LFQ intensities were normalized by dividing the values by the intensity of ScaA1 in CB I, in order to simplify the fold-change analysis between the different samples. A. Identified scaffoldins in each fraction; B. 166 identified dockerin-containing proteins. CB, cellobiose; MCC, microcrystalline cellulose; Doc, dockerin; GH, glycoside hydrolase; CBM, carbohydrate-binding module; CE, carbohydrate esterase; PL, polysaccharide lyases. Significant iBAQ values (≥0.10) are shown in bold. The range of LFQ values is color coded according to the scale shown at the bottom.

**Additional file 5: Figure S2.** Molecular organization of the scaffoldins. Schematic representation of the cohesin-borne scaffoldins. Thirty two scaffoldins of *B. cellulosolvens* possess 79 cohesins that are classified into two main types: type I (33 modules) and type II (43 modules). In addition, Group R was defined for cohesins from scaffoldins ScaR1-R3, whose sequences are notably different than those of the known types. Out of the 32 scaffoldins, 24 were detected in this study. The undetected scaffoldins are represented by gray squares. This Figure was adapted from our previous study [26] with slight changes.

**Additional file 6: Figure S3.** All dockerin-containing proteins detected in *B. cellulosolvens* cells grown on different carbon sources and molecular size fractions. Heatmap of LFQ intensity (log2) of 166 dockerin-containing proteins (see Additional file 4: Table S3B). Zero intensity values were imputed to 10. Rows were standardized, and clustered by partitional clustering using the Euclidian method. Numbers from 1 to 3 at top represent different triplicates from the two substrates: CB, cellobiose, and MCC, microcrystalline cellulose.

**Additional file 7: Table S4.** Mass spectrometry analysis of normalized GH-containing proteins. The intensities of all the proteins in four cellulosomal fractions (CB I, CB II, MCC I and MCC II) were estimated by iBAQ and LFQ methods, in order to evaluate their quantity and abundance. The iBAQ intensities were normalized by dividing each value by that of the major primary scaffoldin ScaA1 in each sample, in order to facilitate interpretation of the results and relate the cellulosomal proteins to the major primary scaffoldin within the samples. The LFQ intensities were normalized by dividing the values by the ScaA1 intensity of CB I in order to simplify the fold-change analysis between the different samples. The table shows 102 identified GH-containing proteins. CB, cellobiose; MCC, microcrystalline cellulose; Doc, dockerin; GH, glycoside hydrolase; CBM, carbohydrate-binding module; CE, carbohydrate esterase; PL, polysaccharide lyases; SLH, S-layer homology module. Significant iBAQ values (≥0.10) are shown in bold.

**Additional file 8: Table S5.** Selected clusters of genes coding CAZyme proteins. The intensities of all the proteins in four cellulosomal fractions (CB I, CB II, MCC I and MCC II) were estimated by iBAQ and LFQ methods in order to evaluate their quantity and abundance. The table shows the expression intensities of clustered proteins represented in Fig. 6.

## Abbreviations

BglC: *Thermobifida fusca* β-glucosidase C
CB: cellobiose
CBM: carbohydrate-binding module
CE: carbohydrate esterase
CMC: carboxymethyl cellulose
Coh: cohesin
CSBM: cell surface-binding module
Doc: dockerin
GH: glycoside hydrolase
iBAQ: intencity based absolute quantification
LFQ: label-free quantification
MCC: microcrystalline cellulose
ORF: open reading frame
PASC: phosphoric acid-swollen cellulose
PCA: principal component analysis
PL: polysaccharide lyase
Sca: scaffoldin
SLH: S-layer homology
VCBS: repeat domain in *Vibrio, Colwellia, Bradyrhizobium* and *Shewanella*
X-Doc: X module coupled with a type II dockerin
Xyn: xylanase
