# Supplementary material for: Unraveling essential cellulosomal components of the (Pseudo)Bacteroides cellulosolvens reveals an extensive reservoir of novel catalytic enzymes
Source: Biotechnol Biofuels. 2019 May 9;12:115. doi: 10.1186/s13068-019-1447-2 (PMC6507058; doi:10.1186/s13068-019-1447-2)
Supplement: Supplementary file 2 — Additional file 2: Figure S1. Chromatographic profile of cellulosomal high-molecular-weight fractions. Gel filtration of supernatant fluids from B. cellulosolvens cells grown on two carbon sources: A, cellobiose and B, microcrystalline cellulose. After concentration, the supernatant fluids were loaded onto a Superose 6 Increase gel filtration column. Two major peaks were obtained during the gel filtration process for both substrates. Examination of the peaks revealed two different populations of high-molecular-weight protein complexes that were active on CMC. The column was calibrated using blue dextran and thyroglobulin. [file 13068_2019_1447_MOESM2_ESM.pdf]

Additional file 2

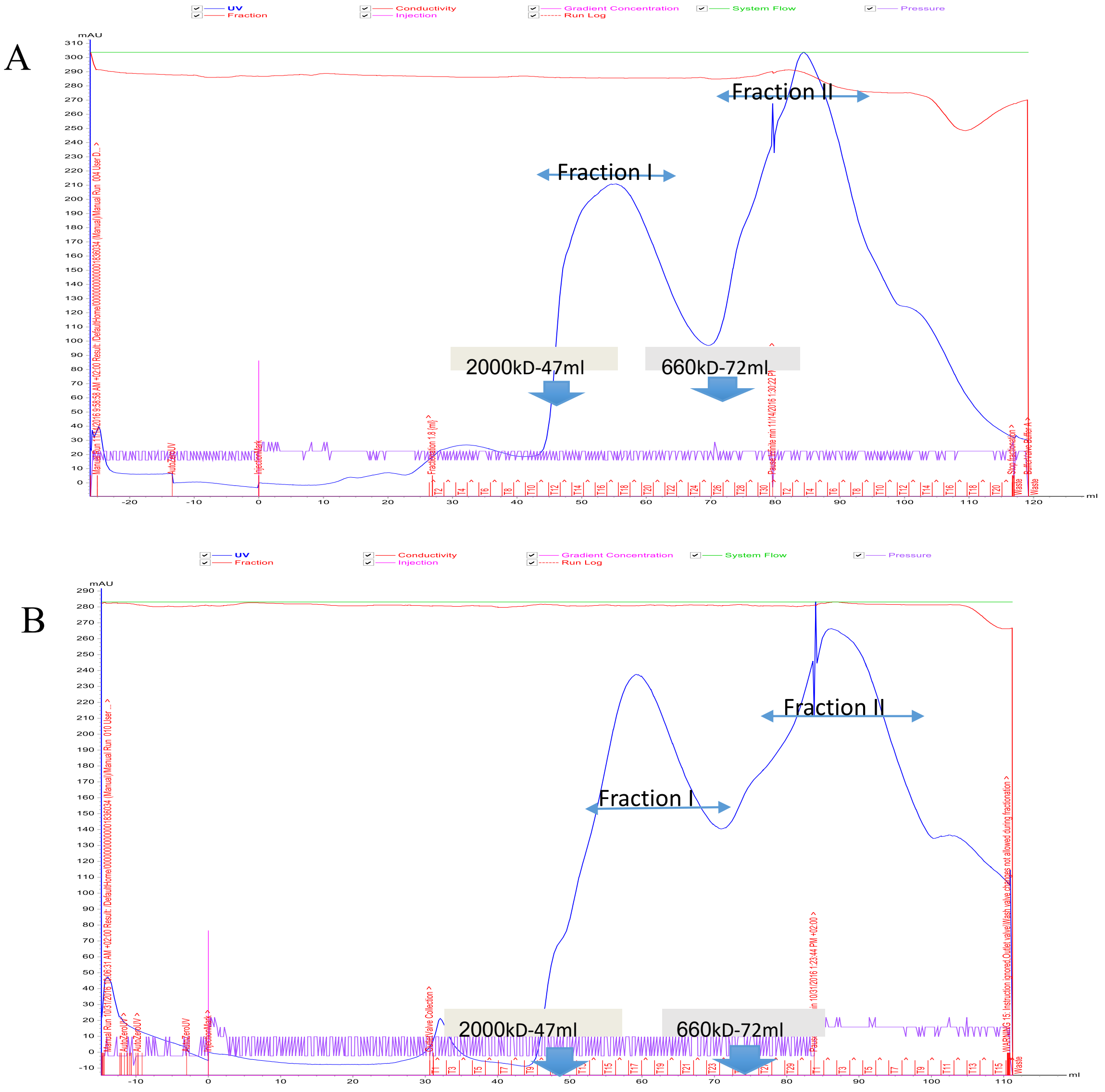

Figure S1. Chromatographic profile of cellulosomal high-molecular-weight fractions. Gel filtration of supernatant fluids from *B. cellulosolvens* cells grown on two carbon sources: A, cellobiose and B, microcrystalline cellulose. After concentration, the supernatant fluids were loaded onto a Superose 6 Increase gel filtration column. Two major peaks were obtained during the gel filtration process for both substrates. Examination of the peaks revealed two different populations of high-molecular-weight protein complexes that were active on CMC. The column was calibrated using blue dextran and thyroglobulin.
