# Supplementary material for: Unraveling essential cellulosomal components of the (Pseudo)Bacteroides cellulosolvens reveals an extensive reservoir of novel catalytic enzymes
Source: Biotechnol Biofuels. 2019 May 9;12:115. doi: 10.1186/s13068-019-1447-2 (PMC6507058; doi:10.1186/s13068-019-1447-2)
Supplement: Supplementary file 4 — Additional file 4: Table S3. Mass spectrometry analysis of normalized cellulosomal proteins. The intensities of all the proteins in four cellulosomal fractions (CB I, CB II, MCC I and MCC II) were estimated by iBAQ and LFQ methods, in order to evaluate their quantity and abundance. The iBAQ intensities were normalized by dividing each value by that of the major primary scaffoldin ScaA1 in each sample, in order to facilitate interpretation of the results and relate the cellulosomal proteins to the major primary scaffoldin within the samples. The LFQ intensities were normalized by dividing the values by the intensity of ScaA1 in CB I, in order to simplify the fold-change analysis between the different samples. A. Identified scaffoldins in each fraction; B. 166 identified dockerin-containing proteins. CB, cellobiose; MCC, microcrystalline cellulose; Doc, dockerin; GH, glycoside hydrolase; CBM, carbohydrate-binding module; CE, carbohydrate esterase; PL, polysaccharide lyases. Significant iBAQ values (≥0.10) are shown in bold. The range of LFQ values is color coded according to the scale shown at the bottom. [file 13068_2019_1447_MOESM4_ESM.pdf]

Additional file 4: Table S3. Mass spectrometry analysis of normalized cellulosomal proteins. The intensities of all the proteins in four cellulosomal fractions (CB I, CB II, MCC I and MCC II) were estimated by iBAQ and LFQ methods, in order to evaluate their quantity and abundance. The iBAQ intensities were normalized by dividing each value by that of the major primary scaffoldin ScaA1 in each sample, in order to facilitate interpretation of the results and relate the cellulosomal proteins to the major primary scaffoldin within the samples. The LFQ intensities were normalized by dividing the values by the intensity of ScaA1 in CB I, in order to simplify the fold-change analysis between the different samples. **A.** Identified scaffoldins in each fraction. **B.** 166 identified dockerin-containing proteins. CB, cellobiose; MCC, microcrystalline cellulose; Doc, dockerin; GH, glycoside hydrolase; CBM, carbohydrate-binding module; CE, carbohydrate esterase; PL, polysaccharide lyases. Significant iBAQ values (≥0.10) are shown in bold. The range of LFQ values is color coded according to the scale shown at the bottom.

A.

		iBAQ Values				LFQ Values			
**Gene name**	**Scaffoldin name**	**iBAQ CB I**	**iBAQ CB II**	**iBAQ MCC I**	**iBAQ MCC II**	**LFQ CB I**	**LFQ CB II**	**LFQ MCC I**	**LFQ MCC II**
Bccel_2050	**ScaA1**	**1.00**	**1.00**	**1.00**	**1.00**	1.000	0.160	1.303	0.302
Bccel_1393	**ScaA2**	0.09	0.02	**0.33**	**0.16**	0.084	0.006	0.516	0.071
Bccel_2049	**ScaB**	0.03	0.02	0.02	0.01	0.033	0.009	0.028	0.010
Bccel_3672	**ScaE**	**1.89**	**3.71**	**1.34**	**2.22**	1.262	0.399	1.261	0.490
Bccel_0335	**ScaF1**	**0.56**	**0.12**	**0.18**	**0.16**	0.139	0.006	0.060	0.012
Bccel_1026	**ScaF2**	0.04	0.04	0.01	0.02	0.010	0.004	0.002	0.003
Bccel_1881	**ScaG**	0.02	**2.79**	0.01	**1.67**	0.008	0.114	0.004	0.154
Bccel_5400	**ScaH2**	**0.12**	**1.87**	**0.29**	**1.62**	0.016	0.047	0.036	0.096
Bccel_3452	**ScaH3**	0.03	0.03	0.01	0.02	0.006	0.003	0.004	0.002
Bccel_1798	**ScaI**	0.00	0.01	0.01	0.03	0.001	0.001	0.004	0.003
Bccel_1025	**ScaJ**	0.01	0.04	0.00	0.02	0.003	0.002	0.001	0.003
Bccel_2276	**ScaL1**	0.04	**0.27**	0.02	**0.20**	0.005	0.013	0.004	0.015
Bccel_2277	**ScaL2**	**0.22**	**0.16**	**0.36**	**0.44**	0.049	0.007	0.094	0.038
Bccel_4693	**ScaM1**	0.03	0.03	0.02	0.02	0.012	0.006	0.011	0.005
Bccel_3957	**ScaM2**	0.00	0.01	0.00	0.01	0.000	0.001	0.000	0.002
Bccel_3191	**ScaO**	0.00	0.00	0.00	0.00	0.001	0.000	0.001	0.001
Bccel_5402	**ScaO2**	0.00	0.03	0.00	0.08	0.001	0.005	0.002	0.015
Bccel_5008	**ScaP**	0.00	0.00	0.00	0.00	0.001	0.000	0.000	0.000
Bccel_1808	**ScaS**	0.06	0.02	0.02	0.01	0.020	0.000	0.007	0.000
Bccel_5396	**ScaT**	0.01	0.05	0.00	0.01	0.001	0.002	0.001	0.001
Bccel_0665	**ScaU**	0.05	0.09	0.01	0.05	0.020	0.010	0.008	0.011
Bccel_1669	**ScaV**	0.00	0.02	0.00	0.01	0.000	0.000	0.000	0.000
Bccel_1027	**ScaW1**	0.01	0.06	0.00	0.01	0.001	0.001	0.001	0.001
Bccel_2278	**ScaW2**	0.00	0.05	0.00	0.02	0.000	0.001	0.000	0.001
					0	0.001	0.01	0.1	1

B.

Gene name	Protein composition	iBAQ CB I	iBAQ CB II	iBAQ MCC I	iBAQ MCC II	LFQ CB I	LFQ CB II	LFQ MCC I	LFQ MCC II
Bccel_2753	GH48-Doc	3.220	7.503	2.983	5.976	1.310	0.467	2.188	0.824
Bccel_3834	CBM4-X229-GH9-Doc	1.795	3.785	1.762	3.899	0.620	0.259	1.066	0.521
Bccel_4718	CBM30-X229-GH9-Doc	0.656	0.385	0.570	0.445	0.418	0.047	0.571	0.105
Bccel_2557	CBM4-X229-GH9-Doc	0.665	0.512	0.727	0.665	0.275	0.052	0.489	0.114
Bccel_1485	GH9-CBM3_1-X60-Doc	0.383	0.374	0.407	0.395	0.229	0.045	0.393	0.089
Bccel_0485	GH9-CBM3-CBM3-X60-Doc	0.237	1.114	0.118	0.831	0.158	0.147	0.139	0.219
Bccel_0913	X60-Doc	0.182	0.123	0.167	0.166	0.146	0.025	0.209	0.059
Bccel_1896	GH9-Doc	0.315	0.147	0.398	0.285	0.117	0.012	0.262	0.046
Bccel_4177	GH30-X92-X60-Doc	0.347	0.158	0.312	0.194	0.107	0.015	0.161	0.032
Bccel_5635	GH74-X60-Doc	0.190	0.127	0.186	0.131	0.103	0.018	0.172	0.033
Bccel_0992	GH9-CBM3_1-X60-Doc	0.223	0.209	0.361	0.338	0.093	0.023	0.280	0.063
Bccel_1632	GH11-CBM6-Doc--GH10	0.221	0.097	0.194	0.133	0.078	0.008	0.139	0.020
Bccel_3943	GH5_1-X60-Doc	0.245	0.242	0.229	0.211	0.078	0.026	0.107	0.041
Bccel_1330	GH9-CBM3_1-X60-Doc	0.161	0.075	0.161	0.152	0.073	0.010	0.123	0.033
Bccel_3527	GH8-Doc	0.357	1.372	0.312	1.076	0.073	0.059	0.109	0.086
Bccel_2462	X60-Doc-CE2	0.211	0.229	0.179	0.229	0.071	0.016	0.092	0.026
Bccel_0290	GH9-CBM3_1-X60-Doc	0.119	0.120	0.158	0.169	0.070	0.019	0.132	0.038
Bccel_3813	GH9-CBM3-X60-Doc	0.153	1.123	0.179	1.025	0.068	0.093	0.135	0.140
Bccel_0820	GH9-CBM3_1-X60-Doc	0.151	0.296	0.163	0.164	0.067	0.035	0.118	0.036
Bccel_2823	GH9-X60-Doc	0.187	0.355	0.206	0.443	0.066	0.026	0.123	0.070
Bccel_1199	X60-Doc-X229-GH9	0.136	0.366	0.316	0.512	0.064	0.038	0.231	0.097
Bccel_1495	GH5_1-X60-Doc	0.147	0.880	0.152	0.475	0.060	0.071	0.114	0.074
Bccel_4152	PL1_5-X60-Doc-PL9_1	0.145	0.047	0.063	0.056	0.059	0.006	0.041	0.012
Bccel_1274	X140-X135-X139-X60-Doc	0.100	0.354	0.127	0.254	0.057	0.046	0.127	0.062
Bccel_1486	CBM22-CE4-CBM22-CBM22-GH10-CBM9-CBM9-X60-DOC	0.057	0.194	0.032	0.137	0.053	0.034	0.052	0.060
Bccel_4932	X60-Doc	0.111	0.172	0.075	0.119	0.052	0.017	0.063	0.022
Bccel_3814	GH10-CBM6-Doc	0.179	0.149	0.237	0.169	0.050	0.012	0.091	0.020
Bccel_1216	GH5-CBM32-X60-Doc	0.134	0.210	0.217	0.250	0.049	0.019	0.101	0.037
Bccel_1260	GH11-Doc	0.227	0.175	0.221	0.193	0.046	0.007	0.078	0.015
Bccel_2493	X140-X135-X139-X60-Doc	0.072	0.112	0.104	0.143	0.045	0.020	0.115	0.046
Bccel_5189	GH9-CBM3_1-Doc	0.089	0.517	0.031	0.248	0.045	0.041	0.028	0.043
Bccel_5136	CBM30-X229-GH9-Doc	0.070	0.178	0.014	0.044	0.042	0.014	0.012	0.010
Bccel_0526	X60-Doc	0.142	0.137	0.266	0.335	0.040	0.012	0.114	0.042
Bccel_5216	CBM4-X229-GH9-Doc	0.080	0.235	0.173	0.464	0.038	0.023	0.143	0.082
Bccel_0895	GH48-Doc	0.078	0.030	0.154	0.071	0.036	0.003	0.117	0.013
Bccel_5539	Doc-cd07761	0.154	0.012	0.135	0.027	0.035	0.001	0.053	0.003
Bccel_5620	CE12-Doc	0.178	0.196	0.117	0.132	0.034	0.011	0.037	0.012
Bccel_5627	PL11_1-Doc	0.101	0.308	0.054	0.285	0.034	0.021	0.036	0.030
Bccel_2002	GH9-X60-Doc	0.095	0.054	0.067	0.049	0.033	0.006	0.042	0.009
Bccel_1280	GH9-CBM3_1-Doc	0.077	0.916	0.075	0.710	0.032	0.086	0.052	0.116
Bccel_0690	GH8-Doc-CE4	0.089	0.025	0.088	0.047	0.032	0.002	0.051	0.009
Bccel_0866	X134-X64-X60-Doc	0.062	0.025	0.053	0.034	0.031	0.004	0.053	0.009
Bccel_4940	X60-Doc	0.103	0.004	0.074	0.004	0.030	0.001	0.034	0.001
Bccel_3616	CBM4-X229-GH9-Doc	0.054	0.089	0.053	0.150	0.029	0.011	0.041	0.025
Bccel_3230	X60-Doc-X188	0.128	0.132	0.082	0.144	0.027	0.005	0.029	0.012
Bccel_4429	pfam05547-X60-Doc	0.077	0.078	0.040	0.057	0.025	0.007	0.024	0.009
Bccel_3733	GH11-GH10-Doc-CE4	0.067	0.071	0.151	0.146	0.024	0.009	0.113	0.034
Bccel_0521	GH9-CBM3-CBM3-X60-Doc	0.038	0.104	0.038	0.083	0.023	0.014	0.039	0.021
Bccel_0991	GH5_1-X60-Doc	0.075	0.052	0.202	0.178	0.021	0.005	0.103	0.025
Bccel_2733	CBM4-X229-GH9-Doc	0.035	0.072	0.125	0.254	0.021	0.010	0.114	0.053
Bccel_0917	CBM35-GH26-Doc	0.093	0.056	0.081	0.065	0.021	0.003	0.031	0.007
Bccel_1286	Doc-CE6	0.106	0.063	0.109	0.079	0.021	0.003	0.039	0.009
Bccel_3614	CBM4-X229-GH9-Doc	0.045	0.069	0.057	0.077	0.020	0.010	0.042	0.018
Bccel_1743	GH30_8-X92-Doc	0.086	0.078	0.056	0.060	0.019	0.005	0.026	0.007
Bccel_0386	GH5_1-Doc	0.075	0.056	0.124	0.117	0.019	0.005	0.055	0.015
Bccel_2822	Doc-GH43	0.051	0.202	0.032	0.179	0.019	0.016	0.021	0.023
Bccel_1738	GH43-CBM13-Doc-GH16	0.032	0.163	0.046	0.091	0.018	0.019	0.041	0.021
Bccel_0535	Doc-X135-X139	0.058	0.023	0.067	0.048	0.018	0.002	0.029	0.006
Bccel_0519	GH9-CBM3_1-X60-Doc	0.044	0.141	0.032	0.094	0.017	0.011	0.023	0.015
Bccel_5541	GH30_5-Doc	0.076	0.021	0.062	0.029	0.017	0.002	0.025	0.004
Bccel_0882	GH9-CBM3_1-X60-Doc	0.051	0.089	0.114	0.075	0.016	0.006	0.053	0.010
Bccel_0881	GH9-CBM3_1-X60-Doc	0.059	0.110	0.105	0.148	0.016	0.006	0.042	0.014
Bccel_2454	GH18-X60-Doc	0.038	0.112	0.048	0.125	0.015	0.011	0.027	0.019
Bccel_5699	X60-Doc	0.031	0.025	0.030	0.058	0.014	0.003	0.024	0.010
Bccel_1250	CBM35-GH26-X60-Doc	0.030	0.129	0.135	0.596	0.014	0.014	0.072	0.072
Bccel_4897	Doc	0.028	0.048	0.065	0.059	0.014	0.008	0.054	0.014
Bccel_2492	GH5_8-GH5_8-Doc	0.021	0.018	0.282	0.338	0.013	0.004	0.276	0.074
Bccel_5617	GH43-X60-Doc	0.068	0.086	0.050	0.043	0.012	0.005	0.019	0.005
Bccel_4009	GH3-X60-Doc	0.024	0.034	0.106	0.117	0.012	0.004	0.085	0.029
Bccel_3613	CBM4-X229-GH9-Doc	0.027	0.061	0.022	0.074	0.011	0.006	0.013	0.013
Bccel_0496	X60-Doc	0.183	0.143	0.104	0.042	0.011	0.002	0.010	0.002
Bccel_5171	X60-Doc-COG5184	0.014	0.117	0.062	0.064	0.010	0.007	0.071	0.024
Bccel_3615	CBM4-X229-GH9-Doc	0.018	0.013	0.016	0.029	0.010	0.002	0.011	0.006
Bccel_5621	X70-CE12-Doc	0.024	0.263	0.025	0.187	0.009	0.017	0.017	0.022
Bccel_1951	CBM3_1-X60-Doc	0.056	0.068	0.038	0.040	0.009	0.003	0.011	0.003
Bccel_4055	X60-Doc	0.022	0.056	0.057	0.097	0.009	0.005	0.034	0.016
Bccel_0520	GH9-CBM3-CBM3-X60-Doc	0.012	0.157	0.012	0.158	0.009	0.026	0.013	0.044
Bccel_5397	Doc-(X159)14	0.016	0.048	0.003	0.011	0.008	0.004	0.004	0.004
Bccel_0571	X60-Doc	0.006	0.006	0.006	0.004	0.008	0.002	0.016	0.004
Bccel_2724	X60-Doc	0.012	0.019	0.012	0.016	0.008	0.004	0.011	0.005
Bccel_5509	Doc	0.077	0.021	0.073	0.061	0.008	0.001	0.010	0.001
Bccel_0923	Doc-(X159)16	0.016	0.009	0.028	0.024	0.007	0.001	0.020	0.004
Bccel_1192	X60-Doc-X295	0.021	0.002	0.023	0.009	0.007	0.000	0.014	0.001

Bccel_1227	X139-X60-Doc	0.020	0.015	0.041	0.024	0.007	0.001	0.026	0.004
Bccel_2113	X260-X60-Doc	0.021	0.032	0.026	0.023	0.007	0.004	0.015	0.004
Bccel_0437	X60-Doc	0.019	**0.125**	0.019	**0.133**	0.007	0.009	0.010	0.014
Bccel_5700	X60-Doc	0.019	0.073	0.007	0.075	0.006	0.006	0.006	0.011
Bccel_3972	Doc	0.002	0.062	0.005	0.002	0.006	0.004	0.009	0.004
Bccel_1242	X140-X135-X139-X60-Doc	0.011	0.005	0.027	0.012	0.006	0.000	0.030	0.005
Bccel_5508	GH5_2-Doc	0.011	**0.147**	0.011	0.058	0.005	0.001	0.010	0.006
Bccel_4973	Doc	0.023	**0.172**	0.020	**0.143**	0.005	0.008	0.010	0.013
Bccel_1750	GH44-X60-Doc	0.016	0.017	0.010	0.017	0.005	0.002	0.006	0.003
Bccel_5100	X260-X60-Doc	0.013	0.099	0.021	0.069	0.005	0.009	0.015	0.014
Bccel_5587	CBM4-X229-GH9-Doc	0.011	0.006	0.016	0.011	0.005	0.001	0.014	0.002
Bccel_3434	X60-Doc-EXPN-CBM63	0.023	0.013	0.028	0.015	0.005	0.001	0.011	0.002
Bccel_2300	X60-Doc	0.009	0.048	0.009	0.056	0.005	0.006	0.007	0.011
Bccel_2732	CBM4-X229-GH9-Doc	0.007	0.024	0.032	**0.170**	0.005	0.005	0.031	0.035
Bccel_3459	COG5184-X60-Doc	0.007	0.058	0.004	0.031	0.004	0.006	0.004	0.006
Bccel_3435	Doc	0.022	0.013	0.020	0.017	0.004	0.001	0.009	0.003
Bccel_0464	X3-X139-Doc	0.013	**0.152**	0.016	**0.126**	0.004	0.009	0.008	0.014
Bccel_3109	CE1-Doc	0.014	0.004	0.006	0.003	0.004	0.000	0.002	0.000
Bccel_0527	Doc-cd01830	0.014	0.063	0.020	0.021	0.004	0.004	0.008	0.002
Bccel_4972	X60-Doc	0.015	0.077	0.044	0.079	0.004	0.004	0.016	0.009
Bccel_0816	X60-Doc	0.015	0.048	0.068	0.055	0.004	0.004	0.023	0.006
Bccel_2186	X60-Doc	0.007	0.010	0.003	0.005	0.004	0.001	0.003	0.001
Bccel_2479	Doc	0.011	0.007	0.009	0.005	0.003	0.000	0.005	0.001
Bccel_5401	CBM6_dist-CBM6_dist-X60-Doc	0.008	0.002	0.005	0.004	0.003	0.000	0.003	0.001
Bccel_4963	X60-Doc	0.009	0.050	0.023	0.093	0.003	0.004	0.013	0.010
Bccel_2734	GH9-Doc	0.028	**0.206**	0.055	**0.522**	0.003	0.004	0.010	0.016
Bccel_3832	X60-Doc-X130	0.004	0.044	0.004	0.052	0.003	0.006	0.004	0.010
Bccel_1672	X60-Doc	0.009	0.052	0.008	0.026	0.003	0.003	0.003	0.004
Bccel_0468	GH81-Doc	0.011	0.007	0.010	0.015	0.003	0.002	0.005	0.008
Bccel_0847	X60-Doc-CE6	0.009	**0.354**	0.023	**0.182**	0.002	0.015	0.011	0.017
Bccel_1770	GH43-CBM13-X60-Doc	0.004	0.050	0.002	0.040	0.002	0.005	0.002	0.008
Bccel_0522	GH9-CBM3_1-X60-Doc	0.005	0.003	0.005	0.003	0.002	0.000	0.004	0.001
Bccel_0394	CBM6-X60-Doc	0.008	0.002	0.003	0.004	0.002	0.000	0.001	0.001
Bccel_5625	X3-X139-Doc	0.004	0.009	0.009	0.020	0.002	0.001	0.005	0.002
Bccel_2471	Doc	0.015	0.023	0.015	0.034	0.002	0.001	0.003	0.003
Bccel_3562	X60-Doc	0.003	0.007	0.038	0.048	0.002	0.001	0.034	0.011
Bccel_0922	X139-CBM4-Doc	0.003	0.041	0.004	**0.121**	0.002	0.003	0.003	0.012
Bccel_0465	GH8-Doc-CE3	0.008	0.000	0.008	0.005	0.002	0.000	0.004	0.001
Bccel_1917	Doc	0.008	0.011	0.008	0.007	0.002	0.001	0.003	0.001
Bccel_5268	X60-Doc-X259	0.009	0.022	0.001	0.005	0.002	0.001	0.001	0.000
Bccel_5217	Doc	0.014	0.021	0.016	0.016	0.002	0.001	0.003	0.002
Bccel_5387	Doc-Doc	0.002	0.064	0.016	**0.222**	0.002	0.006	0.009	0.031
Bccel_5153	GH26-Doc	0.011	0.025	0.007	0.024	0.001	0.001	0.002	0.002
Bccel_0839	Doc-Doc	0.080	**0.186**	0.075	**1.135**	0.001	0.001	0.004	0.015
Bccel_5724	X60-Doc	0.002	0.006	0.004	0.009	0.001	0.000	0.004	0.003
Bccel_5372	COG5184-X60-Doc	0.003	0.021	0.001	0.009	0.001	0.002	0.001	0.002
Bccel_1262	CE3-Doc	0.008	0.003	0.003	0.003	0.001	0.000	0.001	0.000
Bccel_1915	CBM9-Doc	0.004	0.015	0.003	0.016	0.001	0.002	0.002	0.004
Bccel_1660	pfam00149-X60-Doc	0.005	0.009	0.005	0.009	0.001	0.001	0.003	0.002
Bccel_2491	GH5_8-Doc	0.003	0.005	0.070	0.083	0.001	0.000	0.033	0.008
Bccel_4836	X60-Doc	0.004	0.047	0.002	0.015	0.001	0.004	0.001	0.002
Bccel_4135	X60-Doc-X72-X72-X72-X72-X72-FN3-FN3-X83	0.001	0.000	0.001	0.000	0.001	0.000	0.004	0.000
Bccel_4923	X60-Doc	0.005	0.065	0.001	0.036	0.001	0.003	0.000	0.004
Bccel_3097	GH43-CBM6-Doc	0.003	0.001	0.005	0.002	0.001	0.000	0.003	0.000
Bccel_4686	X60-Doc	0.006	0.010	0.006	0.019	0.001	0.001	0.002	0.002
Bccel_3076	GH11-CBM6-Doc-GH10	0.002	0.001	0.003	0.000	0.001	0.000	0.003	0.000
Bccel_1798	X60-Doc	0.003	0.011	0.009	0.023	0.001	0.001	0.004	0.003
Bccel_5725	CE1-Doc	0.004	0.001	0.003	0.003	0.001	0.000	0.001	0.000
Bccel_3093	GH11-GH10-Doc-X124	0.001	0.000	0.002	0.000	0.001	0.000	0.001	0.000
Bccel_1439	cd02619-X135-Doc	0.001	0.005	0.000	0.001	0.001	0.001	0.000	0.000
Bccel_1253	COG5184-X60-Doc	0.001	0.009	0.000	0.005	0.001	0.001	0.001	0.001
Bccel_5179	CE8-CBM35-Doc	0.003	**0.121**	0.015	**0.119**	0.001	0.007	0.009	0.013
Bccel_0518	GH43-Doc	0.005	0.013	0.002	0.008	0.001	0.001	0.000	0.001
Bccel_3151	X60-Doc-PLnc	0.001	0.007	0.003	0.013	0.001	0.001	0.002	0.002
Bccel_4452	Doc-(X159)10	0.001	0.006	0.003	0.014	0.001	0.000	0.001	0.002
Bccel_3095	GH62-CBM6-Doc-GH10	0.001	0.000	0.002	0.001	0.001	0.000	0.002	0.000
Bccel_0545	X60-Doc	0.004	0.018	0.001	0.008	0.000	0.001	0.001	0.001
Bccel_4945	Doc-CBM4-GH16	0.001	0.001	0.001	0.002	0.000	0.000	0.000	0.000
Bccel_1277	X3-Doc	0.000	0.003	0.000	0.015	0.000	0.000	0.000	0.003
Bccel_4653	Doc	0.002	0.000	0.001	0.000	0.000	0.000	0.000	0.000
Bccel_3617	GH9-Doc	0.001	0.000	0.002	0.003	0.000	0.000	0.001	0.000
Bccel_5626	X3-X139-Doc	0.001	0.029	0.002	0.051	0.000	0.002	0.001	0.007
Bccel_4540	X139-Doc	0.001	0.001	0.000	0.000	0.000	0.000	0.000	0.000
Bccel_3077	CE1-Doc	0.002	0.000	0.002	0.001	0.000	0.000	0.000	0.000
Bccel_1771	GH48-Doc	0.001	0.000	0.001	0.001	0.000	0.000	0.000	0.000
Bccel_1455	X60-Doc	0.001	0.001	0.000	0.005	0.000	0.000	0.000	0.000
Bccel_5269	X140-X60-Doc-X259	0.000	0.012	0.001	0.005	0.000	0.000	0.001	0.000
Bccel_2581	pfam09394-Doc	0.000	0.000	0.002	0.003	0.000	0.000	0.001	0.001
Bccel_4869	COG5184-COG5184-X60-Doc	0.000	0.006	0.001	0.030	0.000	0.001	0.002	0.004
Bccel_0689	CE15-CBM6-Doc	0.000	0.000	0.000	0.000	0.000	0.000	0.000	0.000
Bccel_0446	GH8-Doc-CE4	0.000	0.000	0.001	0.000	0.000	0.000	0.000	0.000
Bccel_5276	COG5184-X60-Doc	0.000	0.000	0.001	0.000	0.000	0.000	0.001	0.000
Bccel_4962	X60-Doc	0.000	0.003	0.000	0.003	0.000	0.000	0.000	0.000
					0	0.001	0.01	0.1	1
