# Supplementary material for: Unraveling essential cellulosomal components of the (Pseudo)Bacteroides cellulosolvens reveals an extensive reservoir of novel catalytic enzymes
Source: Biotechnol Biofuels. 2019 May 9;12:115. doi: 10.1186/s13068-019-1447-2 (PMC6507058; doi:10.1186/s13068-019-1447-2)
Supplement: Supplementary file 5 — Additional file 5: Figure S2. Molecular organization of the scaffoldins. Schematic representation of the cohesin-borne scaffoldins. Thirty two scaffoldins of B. cellulosolvens possess 79 cohesins that are classified into two main types: type I (33 modules) and type II (43 modules). In addition, Group R was defined for cohesins from scaffoldins ScaR1-R3, whose sequences are notably different than those of the known types. Out of the 32 scaffoldins, 24 were detected in this study. The undetected scaffoldins are represented by gray squares. This Figure was adapted from our previous study [26] with slight changes. [file 13068_2019_1447_MOESM5_ESM.pdf]

Additional file 5:

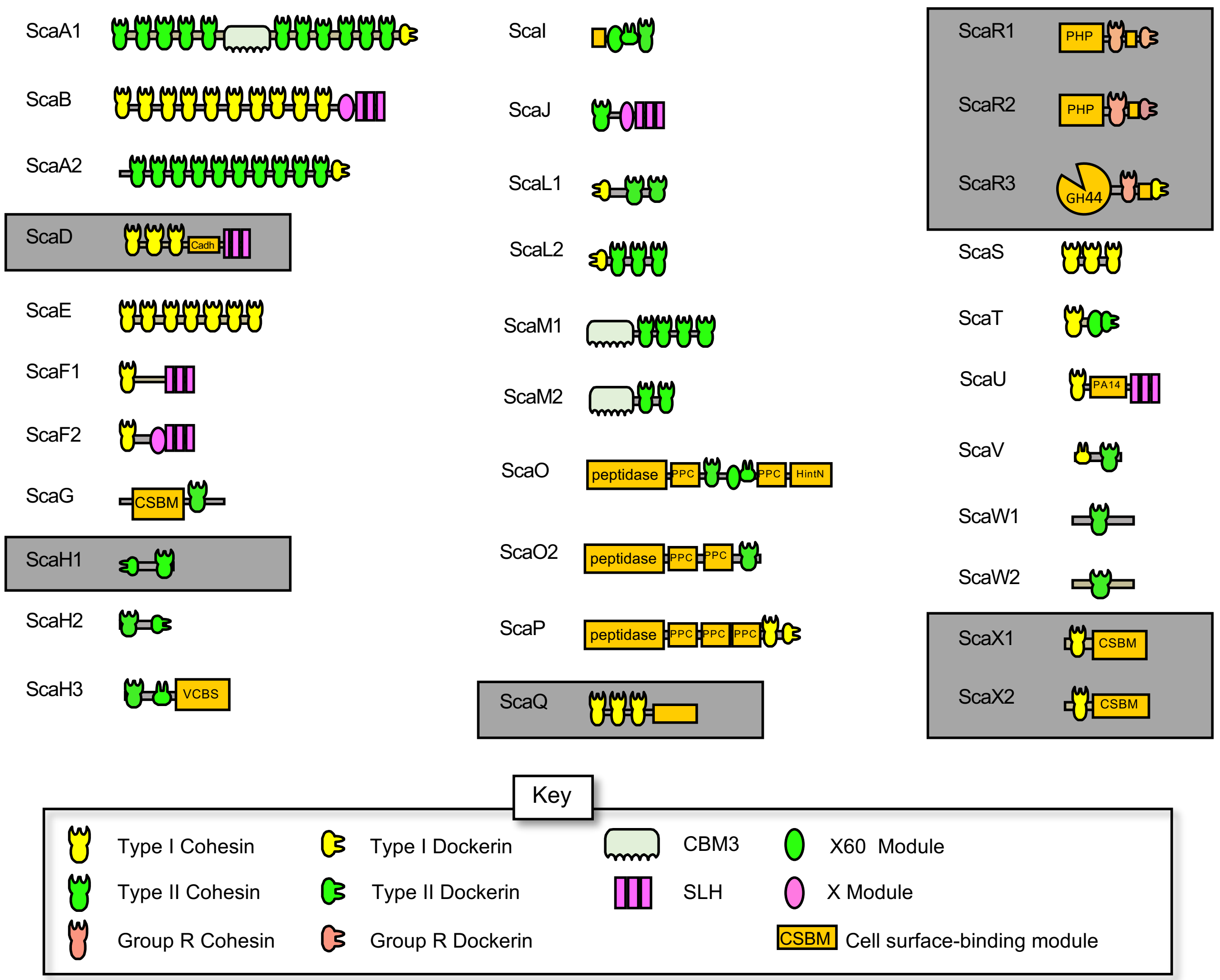

Figure S2: Molecular organization of the scaffoldins.
Schematic representation of the cohesin-borne scaffoldins. Thirty two scaffoldins of *B. cellulosolvens* possess 79 cohesins that are classified into two main types: type I (33 modules) and type II (43 modules). In addition, Group R was defined for cohesins from scaffoldins ScaR1-R3, whose sequences are notably different than those of the known types. Out of the 32 scaffoldins, 24 were detected in this study. The undetected scaffoldins are represented by gray squares. This Figure was adapted from our previous study [Zhivin et al., 2017] with slight changes.

Zhivin O, Dassa B, Morais S, Utturkar SM, Brown SD, Henrissat B, Lamed R, Bayer EA: **Unique organization and unprecedented diversity of the *Bacteroides (Pseudobacteroides) cellulosolvens* cellulosome system.** *Biotechnol Biofuels* 2017, **10:**211.
