# Supplementary material for: Unraveling essential cellulosomal components of the (Pseudo)Bacteroides cellulosolvens reveals an extensive reservoir of novel catalytic enzymes
Source: Biotechnol Biofuels. 2019 May 9;12:115. doi: 10.1186/s13068-019-1447-2 (PMC6507058; doi:10.1186/s13068-019-1447-2)
Supplement: Supplementary file 6 — Additional file 6: Figure S3. All dockerin-containing proteins detected in B. cellulosolvens cells grown on different carbon sources and molecular size fractions. Heatmap of LFQ intensity (log2) of 166 dockerin-containing proteins (see Additional file 4: Table S3B). Zero intensity values were imputed to 10. Rows were standardized, and clustered by partitional clustering using the Euclidian method. Numbers from 1 to 3 at top represent different triplicates from the two substrates: CB, cellobiose, and MCC, microcrystalline cellulose. [file 13068_2019_1447_MOESM6_ESM.pdf]

Additional file 6

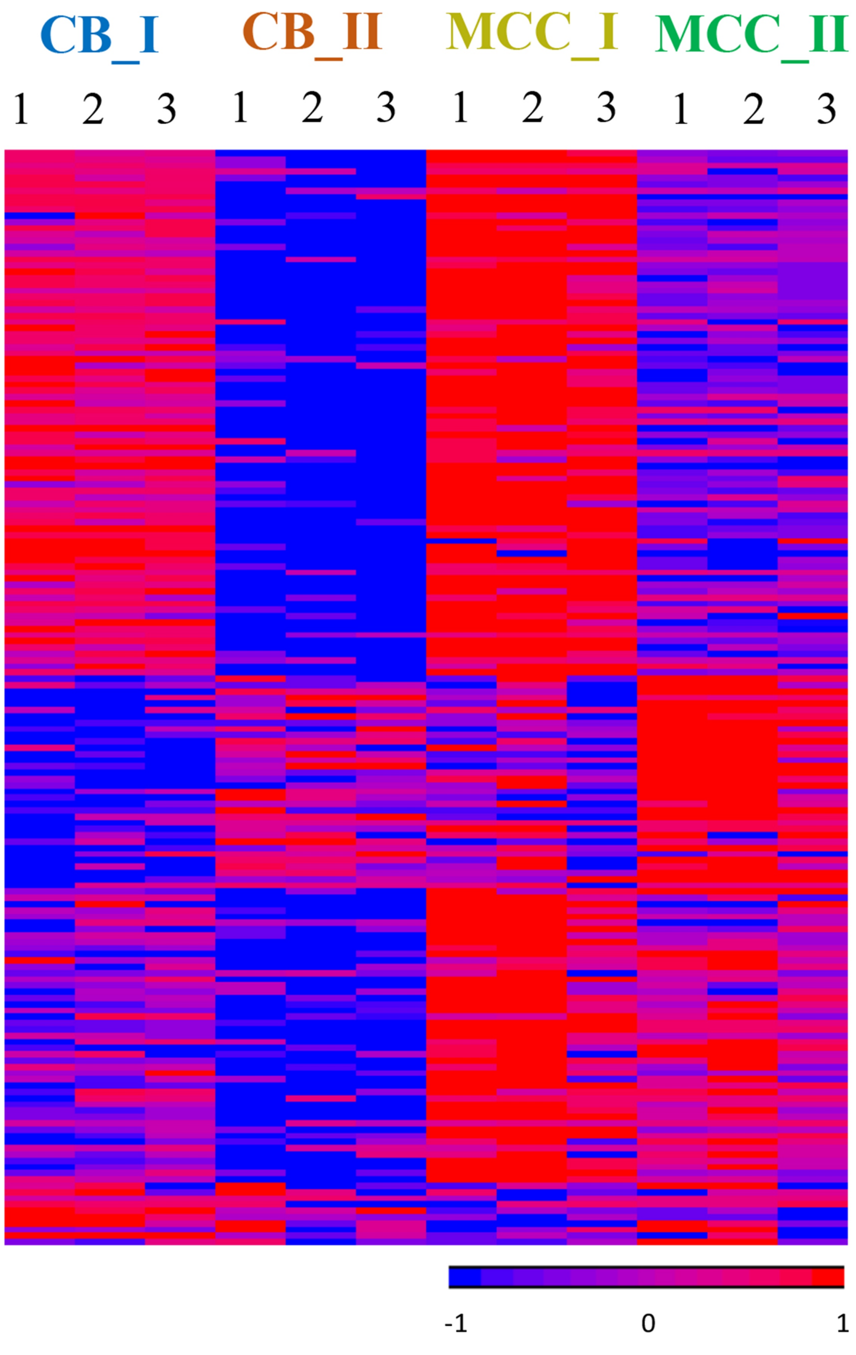

Figure S3: All dockerin-containing proteins detected in *B. cellulosolvens* cells grown on different carbon sources and molecular size fractions.

Heatmap of LFQ intensity (log2) of 166 dockerin-containing proteins (see Additional file 4: Table S3B). Zero intensity values were imputed to 10. Rows were standardized, and clustered by partitional clustering using the Euclidian method. Numbers from 1 to 3 at top represent different triplicates from the two substrates: CB, cellobiose, and MCC, microcrystalline cellulose.
