# Supplementary material for: Unraveling essential cellulosomal components of the (Pseudo)Bacteroides cellulosolvens reveals an extensive reservoir of novel catalytic enzymes
Source: Biotechnol Biofuels. 2019 May 9;12:115. doi: 10.1186/s13068-019-1447-2 (PMC6507058; doi:10.1186/s13068-019-1447-2)
Supplement: Supplementary file 7 — Additional file 7: Table S4. Mass spectrometry analysis of normalized GH-containing proteins. The intensities of all the proteins in four cellulosomal fractions (CB I, CB II, MCC I and MCC II) were estimated by iBAQ and LFQ methods, in order to evaluate their quantity and abundance. The iBAQ intensities were normalized by dividing each value by that of the major primary scaffoldin ScaA1 in each sample, in order to facilitate interpretation of the results and relate the cellulosomal proteins to the major primary scaffoldin within the samples. The LFQ intensities were normalized by dividing the values by the ScaA1 intensity of CB I in order to simplify the fold-change analysis between the different samples. The table shows 102 identified GH-containing proteins. CB, cellobiose; MCC, microcrystalline cellulose; Doc, dockerin; GH, glycoside hydrolase; CBM, carbohydrate-binding module; CE, carbohydrate esterase; PL, polysaccharide lyases; SLH, S-layer homology module. Significant iBAQ values (≥0.10) are shown in bold. [file 13068_2019_1447_MOESM7_ESM.pdf]

Additional file 7

Table S4. Mass spectrometry analysis of normalized GH-containing proteins.
The intensities of all the proteins in four cellulosomal fractions (CB I, CB II, MCC I and MCC II) were estimated by iBAQ and LFQ methods, in order to evaluate their quantity and abundance. The iBAQ intensities were normalized by dividing each value by that of the major primary scaffoldin ScaA1 in each sample, in order to facilitate interpretation of the results and relate the cellulosomal proteins to the major primary scaffoldin within the samples. The LFQ intensities were normalized by dividing the values by the ScaA1 intensity of CB I in order to simplify the fold-change analysis between the different samples. The table shows 102 identified GH-containing proteins. CB, cellobiose; MCC, microcrystalline cellulose; Doc, dockerin; GH, glycoside hydrolase; CBM, carbohydrate-binding module; CE, carbohydrate esterase; PL, polysaccharide lyases**;** SLH, S-layer homology module. Significant iBAQ values (≥0.10) are shown in bold.

Gene name	Protein composition	iBAQ CB I	iBAQ CB II	iBAQ MCC I	iBAQ MCC II	LFQ CB I	LFQ CB II	LFQ MCC I	LFQ MCC II
Bccel_0290	GH9-CBM3_1-X60-Doc	**0.119**	**0.120**	**0.158**	**0.169**	0.070	0.019	0.132	0.038
Bccel_0386	GH5_1-Doc	0.075	0.056	**0.124**	**0.117**	0.019	0.005	0.055	0.015
Bccel_0446	GH8-Doc-CE4	0.000	0.000	0.001	0.000	0.000	0.000	0.000	0.000
Bccel_0465	GH8-Doc-CE3	0.008	0.000	0.008	0.005	0.002	0.000	0.004	0.001
Bccel_0468	GH81-Doc	0.011	0.007	0.010	0.015	0.003	0.002	0.005	0.008
Bccel_0485	GH9-CBM3-CBM3-X60-Doc	**0.237**	**1.114**	**0.118**	**0.831**	0.158	0.147	0.139	0.219
Bccel_0497	GH53	**0.142**	**0.136**	0.088	**0.120**	0.035	0.007	0.040	0.014
Bccel_0518	GH43-Doc	0.005	0.013	0.002	0.008	0.001	0.001	0.000	0.001
Bccel_0519	GH9-CBM3_1-X60-Doc	0.044	**0.141**	0.032	0.094	0.017	0.011	0.023	0.015
Bccel_0520	GH9-CBM3-CBM3-X60-Doc	0.012	**0.157**	0.012	**0.158**	0.009	0.026	0.013	0.044
Bccel_0521	GH9-CBM3-CBM3-X60-Doc	0.038	**0.104**	0.038	0.083	0.023	0.014	0.039	0.021
Bccel_0522	GH9-CBM3_1-X60-Doc	0.005	0.003	0.005	0.003	0.002	0.000	0.004	0.001
Bccel_0690	GH8-Doc-CE4	0.089	0.025	0.088	0.047	0.032	0.002	0.051	0.009
Bccel_0723	GH5_4	0.001	0.005	0.000	0.019	0.001	0.001	0.001	0.001
Bccel_0820	GH9-CBM3_1-X60-Doc	**0.151**	**0.296**	**0.163**	**0.164**	0.067	0.035	0.118	0.036
Bccel_0881	GH9-CBM3_1-X60-Doc	0.059	**0.110**	**0.105**	**0.148**	0.016	0.006	0.042	0.014
Bccel_0882	GH9-CBM3_1-X60-Doc	0.051	0.089	**0.114**	0.075	0.016	0.006	0.053	0.010
Bccel_0895	GH48-Doc	0.078	0.030	**0.154**	0.071	0.036	0.003	0.117	0.013
Bccel_0904	GH94-X91	0.064	**0.421**	0.013	**0.169**	0.038	0.045	0.018	0.042
Bccel_0905	GH3	0.000	0.082	0.000	0.027	0.000	0.006	0.000	0.004
Bccel_0909	CBM4-X229-GH9	0.081	0.046	0.056	0.077	0.038	0.009	0.045	0.023
Bccel_0917	CBM35-GH26-Doc	0.093	0.056	0.081	0.065	0.021	0.003	0.031	0.007
Bccel_0991	GH5_1-X60-Doc	0.075	0.052	**0.202**	**0.178**	0.021	0.005	0.103	0.025
Bccel_0992	GH9-CBM3_1-X60-Doc	**0.223**	**0.209**	**0.361**	**0.338**	0.093	0.023	0.280	0.063
Bccel_1103	CBM4-X229-GH9	0.001	0.000	0.001	0.001	0.000	0.000	0.000	0.000
Bccel_1108	GH11-CBM6-CBM6-CE4	0.004	0.005	0.001	0.002	0.001	0.000	0.000	0.000
Bccel_1199	X60-Doc-X229-GH9	**0.136**	**0.366**	**0.316**	**0.512**	0.064	0.038	0.231	0.097
Bccel_1216	GH5-CBM32-X60-Doc	**0.134**	**0.210**	**0.217**	**0.250**	0.049	0.019	0.101	0.037
Bccel_1250	CBM35-GH26-X60-Doc	0.030	**0.129**	**0.135**	**0.596**	0.014	0.014	0.072	0.072
Bccel_1260	GH11-Doc	**0.227**	**0.175**	**0.221**	**0.193**	0.046	0.007	0.078	0.015
Bccel_1280	GH9-CBM3_1-Doc	0.077	**0.916**	0.075	**0.710**	0.032	0.086	0.052	0.116
Bccel_1330	GH9-CBM3_1-X60-Doc	**0.161**	0.075	**0.161**	**0.152**	0.073	0.010	0.123	0.033

Bccel_1373	GH25	0.001	0.000	0.000	0.000	0.000	0.000	0.000	0.000
Bccel_1425	GH10	0.000	0.005	0.000	0.003	0.000	0.000	0.000	0.000
Bccel_1485	GH9-CBM3_1-X60-Doc	0.383	0.374	0.407	0.395	0.229	0.045	0.393	0.089
Bccel_1486	CBM22-CE4-CBM22-CBM22-GH10-CBM9-CBM9-X60-Doc	0.057	0.194	0.032	0.137	0.053	0.034	0.052	0.060
Bccel_1491	CBM22-CE4-CBM22-GH10-CBM9-CBM9-SLH-SLH-SLH	0.210	1.342	0.068	1.009	0.187	0.232	0.106	0.318
Bccel_1495	GH5_1-X60-Doc	0.147	0.880	0.152	0.475	0.060	0.071	0.114	0.074
Bccel_1632	GH11-CBM6-Doc--GH10	0.221	0.097	0.194	0.133	0.078	0.008	0.139	0.020
Bccel_1712	GH43	0.004	0.397	0.002	0.152	0.001	0.013	0.001	0.010
Bccel_1738	GH43-CBM13-Doc-GH16	0.032	0.163	0.046	0.091	0.018	0.019	0.041	0.021
Bccel_1743	GH30_8-X92-Doc	0.086	0.078	0.056	0.060	0.019	0.005	0.026	0.007
Bccel_1750	GH44-X60-Doc	0.016	0.017	0.010	0.017	0.005	0.002	0.006	0.003
Bccel_1770	GH43-CBM13-X60-Doc	0.004	0.050	0.002	0.040	0.002	0.005	0.002	0.008
Bccel_1771	GH48-Doc	0.001	0.000	0.001	0.001	0.000	0.000	0.000	0.000
Bccel_1896	GH9-Doc	0.315	0.147	0.398	0.285	0.117	0.012	0.262	0.046
Bccel_1950	GH9	0.052	0.053	0.033	0.030	0.015	0.005	0.018	0.005
Bccel_2002	GH9-X60-Doc	0.095	0.054	0.067	0.049	0.033	0.006	0.042	0.009
Bccel_2005	GH94-X91	0.025	0.539	0.021	0.159	0.018	0.056	0.021	0.039
Bccel_2133	GH2	0.001	0.001	0.001	0.001	0.000	0.000	0.001	0.000
Bccel_2454	GH18-X60-Doc	0.038	0.112	0.048	0.125	0.015	0.011	0.027	0.019
Bccel_2491	GH5_8-Doc	0.003	0.005	0.070	0.083	0.001	0.000	0.033	0.008
Bccel_2492	GH5_8-GH5_8-Doc	0.021	0.018	0.282	0.338	0.013	0.004	0.276	0.074
Bccel_2557	CBM4-X229-GH9-Doc	0.665	0.512	0.727	0.665	0.275	0.052	0.489	0.114
Bccel_2732	CBM4-X229-GH9-Doc	0.007	0.024	0.032	0.170	0.005	0.005	0.031	0.035
Bccel_2733	CBM4-X229-GH9-Doc	0.035	0.072	0.125	0.254	0.021	0.010	0.114	0.053
Bccel_2734	GH9-Doc	0.028	0.206	0.055	0.522	0.003	0.004	0.010	0.016
Bccel_2735	CBM4-X229-GH9	0.025	0.197	0.063	0.588	0.011	0.019	0.040	0.106
Bccel_2753	GH48-Doc	3.220	7.503	2.983	5.976	1.310	0.467	2.188	0.824
Bccel_2822	Doc-GH43	0.051	0.202	0.032	0.179	0.019	0.016	0.021	0.023
Bccel_2823	GH9-X60-Doc	0.187	0.355	0.206	0.443	0.066	0.026	0.123	0.070
Bccel_3064	GH10-GH10-SLH	0.006	0.160	0.003	0.193	0.010	0.035	0.010	0.078
Bccel_3076	GH11-CBM6-Doc-GH10	0.002	0.001	0.003	0.000	0.001	0.000	0.003	0.000
Bccel_3093	GH11-GH10-Doc-X124	0.001	0.000	0.002	0.000	0.001	0.000	0.001	0.000
Bccel_3095	GH62-CBM6-Doc-GH10	0.001	0.000	0.002	0.001	0.001	0.000	0.002	0.000
Bccel_3097	GH43-CBM6-Doc	0.003	0.001	0.005	0.002	0.001	0.000	0.003	0.000
Bccel_3298	GH3	0.000	0.001	0.000	0.002	0.000	0.000	0.000	0.000
Bccel_3299	GH67	0.008	0.001	0.007	0.003	0.003	0.001	0.006	0.001
Bccel_3527	GH8-Doc	0.357	1.372	0.312	1.076	0.073	0.059	0.109	0.086
Bccel_3613	CBM4-X229-GH9-Doc	0.027	0.061	0.022	0.074	0.011	0.006	0.013	0.013
Bccel_3614	CBM4-X229-GH9-Doc	0.045	0.069	0.057	0.077	0.020	0.010	0.042	0.018
Bccel_3615	CBM4-X229-GH9-Doc	0.018	0.013	0.016	0.029	0.010	0.002	0.011	0.006
Bccel_3616	CBM4-X229-GH9-Doc	0.054	0.089	0.053	0.150	0.029	0.011	0.041	0.025
Bccel_3617	GH9-Doc	0.001	0.000	0.002	0.003	0.000	0.000	0.001	0.000
Bccel_3618	CBM4-X229-GH9	0.013	0.027	0.018	0.028	0.004	0.002	0.007	0.004
Bccel_3733	GH11-GH10-Doc-CE4	0.067	0.071	0.151	0.146	0.024	0.009	0.113	0.034
Bccel_3813	GH9-CBM3-X60-Doc	0.153	1.123	0.179	1.025	0.068	0.093	0.135	0.140
Bccel_3814	GH10-CBM6-Doc	0.179	0.149	0.237	0.169	0.050	0.012	0.091	0.020
Bccel_3834	CBM4-X229-GH9-Doc	1.795	3.785	1.762	3.899	0.620	0.259	1.066	0.521
Bccel_3943	GH5_1-X60-Doc	0.245	0.242	0.229	0.211	0.078	0.026	0.107	0.041
Bccel_4009	GH3-X60-Doc	0.024	0.034	0.106	0.117	0.012	0.004	0.085	0.029
Bccel_4126	GH3	0.000	0.004	0.000	0.001	0.000	0.000	0.000	0.000
Bccel_4177	GH30-X92-X60-Doc	0.347	0.158	0.312	0.194	0.107	0.015	0.161	0.032
Bccel_4191	CBM2-GH5_1	0.000	0.000	0.000	0.000	0.000	0.000	0.000	0.000
Bccel_4498	GH5_37	0.001	0.089	0.000	0.059	0.000	0.004	0.000	0.005
Bccel_4519	CBM50-GH18-pfam07833	0.028	0.232	0.008	0.086	0.013	0.021	0.008	0.018
Bccel_4718	CBM30-X229-GH9-Doc	0.656	0.385	0.570	0.445	0.418	0.047	0.571	0.105
Bccel_4945	Doc-CBM4-GH16	0.001	0.001	0.001	0.002	0.000	0.000	0.000	0.000
Bccel_5136	CBM30-X229-GH9-Doc	0.070	0.178	0.014	0.044	0.042	0.014	0.012	0.010
Bccel_5153	GH26-Doc	0.011	0.025	0.007	0.024	0.001	0.001	0.002	0.002
Bccel_5189	GH9-CBM3_1-Doc	0.089	0.517	0.031	0.248	0.045	0.041	0.028	0.043
Bccel_5216	CBM4-X229-GH9-Doc	0.080	0.235	0.173	0.464	0.038	0.023	0.143	0.082
Bccel_5320	GH3	0.002	0.116	0.000	0.039	0.001	0.011	0.000	0.009
Bccel_5508	GH5_2-Doc	0.011	0.147	0.011	0.058	0.005	0.001	0.010	0.006
Bccel_5538	GH115	0.000	0.001	0.000	0.001	0.000	0.000	0.000	0.000
Bccel_5541	GH30_5-Doc	0.076	0.021	0.062	0.029	0.017	0.002	0.025	0.004
Bccel_5587	CBM4-X229-GH9-Doc	0.011	0.006	0.016	0.011	0.005	0.001	0.014	0.002
Bccel_5603	GH10-CBM9_dist-CBM9_dist	0.001	0.003	0.000	0.000	0.000	0.000	0.000	0.000
Bccel_5617	GH43-X60-Doc	0.068	0.086	0.050	0.043	0.012	0.005	0.019	0.005
Bccel_5635	GH74-X60-Doc	0.190	0.127	0.186	0.131	0.103	0.018	0.172	0.033
Bccel_2759	X104-CBM48-GH13_9	0.001	0.027	0.000	0.011	0.000	0.003	0.000	0.002
Bccel_4024	GH18	0.000	0.001	0.000	0.001	0.000	0.000	0.000	0.000
					0	0.001	0.01	0.1	1
