# Supplementary material for: Unraveling essential cellulosomal components of the (Pseudo)Bacteroides cellulosolvens reveals an extensive reservoir of novel catalytic enzymes
Source: Biotechnol Biofuels. 2019 May 9;12:115. doi: 10.1186/s13068-019-1447-2 (PMC6507058; doi:10.1186/s13068-019-1447-2)
Supplement: Supplementary file 8 — Additional file 8: Table S5. Selected clusters of genes coding CAZyme proteins. The intensities of all the proteins in four cellulosomal fractions (CB I, CB II, MCC I and MCC II) were estimated by iBAQ and LFQ methods in order to evaluate their quantity and abundance. The table shows the expression intensities of clustered proteins represented in Fig. 6. [file 13068_2019_1447_MOESM8_ESM.pdf]

Additional file 8: Table S5. Selected clusters of genes coding CAZyme proteins.
The intensities of all the proteins in four cellulosomal fractions (CB I, CB II, MCC I and MCC II) were estimated by iBAQ and LFQ methods in order to evaluate their quantity and abundance. The table shows the expression intensities of clustered proteins represented in Figure 6.

Gene name	Protein composition	iBAQ CB I	iBAQ CB II	iBAQ MCC I	iBAQ MCC II	LFQ CB I	LFQ CB II	LFQ MCC I	LFQ MCC II
Bccel_0518	GH43-Doc	0.005	0.013	0.002	0.008	0.001	0.001	0.000	0.001
Bccel_0519	GH9-CBM3_1-X60-Doc	0.044	**0.141**	0.032	0.094	0.017	0.011	0.023	0.015
Bccel_0520	GH9-CBM3-CBM3-X60-Doc	0.012	**0.157**	0.012	**0.158**	0.009	0.026	0.013	0.044
Bccel_0521	GH9-CBM3-CBM3-X60-Doc	0.038	**0.104**	0.038	0.083	0.023	0.014	0.039	0.021
Bccel_0522	GH9-CBM3_1-X60-Doc	0.005	0.003	0.005	0.003	0.002	0.000	0.004	0.001
Bccel_0526	X60-Doc	**0.142**	**0.137**	**0.266**	**0.335**	0.040	0.012	0.114	0.042
Bccel_0527	Doc-cd01830	0.014	0.063	0.020	0.021	0.004	0.004	0.008	0.002
Bccel_0904	GH94-X91	0.064	**0.421**	0.013	**0.169**	0.038	0.045	0.018	0.042
Bccel_0905	GH3	0.000	0.082	0.000	0.027	0.000	0.006	0.000	0.004
Bccel_0909	CBM4-X229-GH9	0.081	0.046	0.056	0.077	0.038	0.009	0.045	0.023
Bccel_0913	X60-Doc	**0.182**	**0.123**	**0.167**	**0.166**	0.146	0.025	0.209	0.059
Bccel_0917	CBM35-GH26-Doc	0.093	0.056	0.081	0.065	0.021	0.003	0.031	0.007
Bccel_0922	X139-CBM4-Doc	0.003	0.041	0.004	**0.121**	0.002	0.003	0.003	0.012
Bccel_0923	Doc-(X159)16	0.016	0.009	0.028	0.024	0.007	0.001	0.020	0.004
Bccel_2732	CBM4-X229-GH9-Doc	0.007	0.024	0.032	**0.170**	0.005	0.005	0.031	0.035
Bccel_2733	CBM4-X229-GH9-Doc	0.035	0.072	**0.125**	**0.254**	0.021	0.010	0.114	0.053
Bccel_2734	GH9-Doc	0.028	**0.206**	0.055	**0.522**	0.003	0.004	0.010	0.016
Bccel_2735	CBM4-X229-GH9	0.025	**0.197**	0.063	**0.588**	0.011	0.019	0.040	0.106
Bccel_3613	CBM4-X229-GH9-Doc	0.027	0.061	0.022	0.074	0.011	0.006	0.013	0.013
Bccel_3614	CBM4-X229-GH9-Doc	0.045	0.069	0.057	0.077	0.020	0.010	0.042	0.018
Bccel_3615	CBM4-X229-GH9-Doc	0.018	0.013	0.016	0.029	0.010	0.002	0.011	0.006
Bccel_3616	CBM4-X229-GH9-Doc	0.054	0.089	0.053	**0.150**	0.029	0.011	0.041	0.025
Bccel_3617	GH9-Doc	0.001	0.000	0.002	0.003	0.000	0.000	0.001	0.000
Bccel_3618	CBM4-X229-GH9	0.013	0.027	0.018	0.028	0.004	0.002	0.007	0.004
Bccel_5620	CE12-Doc	0.178	0.196	0.117	0.132	0.034	0.011	0.037	0.012
Bccel_5621	X70-CE12-Doc	0.024	0.263	0.025	0.187	0.009	0.017	0.017	0.022
Bccel_5625	X3-X139-Doc	0.004	0.009	0.009	0.020	0.002	0.001	0.005	0.002
Bccel_5626	X3-X139-Doc	0.001	0.029	0.002	0.051	0.000	0.002	0.001	0.007
Bccel_5627	PL11_1-Doc	0.101	0.308	0.054	0.285	0.034	0.021	0.036	0.030
					0	0.001	0.01	0.1	1
